# Tenets in Microbial Endocrinology: A New Vista in Teleost Reproduction

**DOI:** 10.3389/fphys.2022.871045

**Published:** 2022-08-12

**Authors:** Ramjanul Haque, Ipsita Iswari Das, Paramita Banerjee Sawant, Narinder Kumar Chadha, Lakshman Sahoo, Rajesh Kumar, Jitendra Kumar Sundaray

**Affiliations:** ^1^ Division of Aquaculture, ICAR-Central Institute of Fisheries Education, Mumbai, India; ^2^ Fish Genetics and Biotechnology Division, ICAR-Central Institute of Freshwater Aquaculture, Bhubaneswar, India; ^3^ Aquaculture Production and Environment Division, ICAR-Central Institute of Freshwater Aquaculture, Bhubaneswar, India

**Keywords:** aquatic organisms, microbial composition, microbiota-gut-brain axis, neurohormone, neuroactive metabolites, physiology, aquaculture

## Abstract

Climate vulnerability and induced changes in physico-chemical properties of aquatic environment can bring impairment in metabolism, physiology and reproduction in teleost. Variation in environmental stimuli mainly acts on reproduction by interfering with steroidogenesis, gametogenesis and embryogenesis. The control on reproductive function in captivity is essential for the sustainability of aquaculture production. There are more than 3,000 teleost species across the globe having commercial importance; however, adequate quality and quantity of seed production have been the biggest bottleneck. Probiotics are widely used in aquaculture as a growth promoter, stress tolerance, pathogen inhibition, nutrient digestibility and metabolism, reproductive performance and gamete quality. As the gut microbiota exerts various effects on the intestinal milieu which influences distant organs and pathways, therefore it is considered to be a full-fledged endocrine organ. Researches on Gut-Brain-Gonad axis (GBG axis) and its importance on physiology and reproduction have already been highlighted for higher mammals; however, the study on fish physiology and reproduction is limited. While looking into the paucity of information, we have attempted to review the present status of microbiome and its interaction between the brain and gut. This review will address a process of the microbiome physiological mechanism involved in fish reproduction. The gut microbiota influences the BPG axis through a wide variety of compounds, including neuropeptides, neurotransmitter homologs and transmitters. Currently, research is being conducted to determine the precise process by which gut microbial composition influences brain function in fish. The gut-brain bidirectional interaction can influence brain biochemistry such as GABA, serotonin and tryptophan metabolites which play significant roles in CNS regulation. This review summarizes the fact, how microbes from gut, skin and other parts of the body influence fish reproduction through the Gut-Brain-Gonad axis.

## Introduction

Aquaculture is the fastest expanding agricultural sector, accounting for over half of all seafood (FAO, 2020) and is frequently promoted as a solution for fulfilling the century’s growing food demands. Presently, about 424 aquatic species are cultivated globally, providing nourishment, food security, and livelihoods to millions of people (Barange et al., 2018). In 2016, around 59.6 million individuals worked in the primary sector of capture fisheries and aquaculture, with aquaculture accounting for 32% of this total (Bhari and Visvanathan, 2018). In 2016, China, India, Indonesia, Vietnam, and Bangladesh accounted for 82.2 % of global production by quantity (FAO, 2018). According to this viewpoint, aquaculture has received significant scholarly attention, including the recent IPCC 1.5°C report (Tollefson, 2018), which identifies aquaculture as one of the key sectors that require attention on global food security and the upgrading of adaptation policy. Aquaculture systems undergoing massive changes, like other widely researched resource systems in climate adaptation research, should be able to respond innovatively in order to adapt more rapidly and completely to mitigate obstacles and thrive potential possibilities (Siders, 2019). Fish physiology and their reproductive performances in likely to be affected by increasing water temperatures arising from climate changes. Rapid fluctuations in environmental factors causes’ negative impact on fish breeding, hatching and larval survivability. The role of endogenous microbiota in fish reproduction, on the other hand, has received little attention. Understanding the pathways of microbiota-gut-brain linkages in reproductive biology, endocrinology, and gonadal physiology will aid in captive maturation, effective breeding, and seed production. Nonetheless, while microbiologists study microbiota-gut-brain interactions in behavior and recognize such intricacies, the equivalent complexity of the host neurophysiological system, especially within the gut is usually neglected (Lyte, 2014). The reproductive microbiome has been found to create selective pressures on males and females, with severe implications for sexual selection, conflict, mating systems, and reproductive barriers. According to microbial endocrinology, the microbiome may influence teleost social behaviour, sex differentiation, and sex determination.

## Microbiome Composition in Teleost

Microorganisms are vital for animal survival and physiological functions (McFall-Ngai et al., 2013). Different anatomical niches (e.g., skin, reproductive tract, *etc.*) of an organism have distinct microbiomes, but the vast majority of microbes inhabits the gastrointestinal (GI) tract and plays a censorious role in a multiple way of physiological processes. The term “gut microbiota” refers to the hundreds of billions of complex assemblages of bacteria found in the digestive tracts of vertebrates including fish. Betaproteobacteria (*Janthinobacterium* and *Rhodoferax*) are the dominating bacteria in fish eggs (Ghanbari et al., 2015). The most prevalent bacteria in the GI tract during the first feeding stages are *Shewanella* and *Aeromonas* spp, and in juveniles weighing more than 2g are *Pseudomonas* and *Aeromonas* spp. (Romero and Navarrete, 2006). The fish microbiome is diversified, containing protists, fungi, yeasts, viruses, and members of bacteria and archaea (Merrifield and Rodiles, 2015). Approximately 500 distinct kinds of bacteria have been found in the fish GI tract, which is dominated by aerobes or facultative anaerobes as well as stringent anaerobes (Legrand et al., 2020). Rawls et al. (2006) discovered that Proteobacteria, along with Bacteroidetes and Firmicutes, make up 90 percent of the microbiota in the digestive tracts of several fish species (Ghanbari et al., 2015), and Fusobacteria, Actinobacteria, Clostridia, Bacilli, and Verrucomicrobia are among the most represented phyla (Givens et al., 2015). In addition, available literatures have been reviewed and reported in Supplementary Table S1. The microbial population, composition, and function of the fish gut differ in different parts (Clements et al., 2014). For a species the salinity of its habitat, its trophic level, and its taxonomy all have a strong correlation with microbial diversity. Furthermore, the microbial community is divided into two primary groups: allochthonous (free-living, transient microbiota) and autochthonous (microbiota colonise the mucosal surface of the digestive system), the latter of which constitutes the core population in vertebrates (Nayak, 2010; Banerjee and Ray, 2017). The composition varies as a consequence of natural environment. Freshwater specie’s guts are dominated by *Acinetobacter*, *Aeromonas*, *Flavobacterium, Lactococcus*, and *Pseudomonas,* as well as obligate anaerobes such as *Bacteroides*, *Clostridium*, and *Fusobacterium*, and members of the Enterobacteriaceae family (Gómez and Balcázar, 2008). *Aeromonas*, *Alcaligenes, Alteromonas, Carnobacterium, Flavobacterium, Micrococcus, Moraxella, Pseudomonas*, and *Vibrio* dominate the intestines of marine fish (Gómez and Balcázar, 2008). The genus *Vibrio* that contains both dangerous and probiotic (health-promoting) species (Vandenberghe et al., 2003), is one of the most important bacterial genera in aquaculture. *Vibrio alginolyticus* acts as a probiotic for Atlantic salmon, protecting it against *Aeromonas salmonicida*, *Vibrio anguillarum*, and *Vibrio ordalii* (Kim et al., 2007; Yan and Chen, 2015). The most common bacterial diseases of marine fish and invertebrates are *V. anguillarum, V. salmonicida, and V. vulnificus* (Kim et al., 2007). According to Thompson et al. (2004), many *Vibrio* species are function as symbionts, releasing hydrolytic enzymes that aid in the digestion of food components. *Photobacterium iliopiscarium*, a non-luminescent bacterium, was obtained from the intestines of cold-water fishes (Onarheim et al., 1994; Urakawa et al., 1999). Numerous *Photobacterium* aid in chitin digestion in the host stomach (MacDonald et al., 1986; Itoi et al., 2006). The density of enzyme-producing bacteria in the gastrointestinal tract (GI tract) of four brackish water teleosts (*Scatophagus argus, Terapon jarbua, Mystus gulio*, and *Etroplus suratensis*) revealed that the density increases with GIT length (Fidopiastis et al., 2006; Bakke-McKellep et al., 2007; Silva et al., 2011; Hovda et al., 2012; Das et al., 2014). Previously, most studies were conducted on mammals, but now some researchers are focusing on elucidating the role of microbiota (present in the gill, gut, intestine, and skin regions) in aquatic organisms, particularly in fish. The role of the gut microbiota in aquatic organisms appears similar to that in terrestrial animals and strengthens the digestive and immune systems (Talwar et al., 2018). These complex microbial communities, interact with each other as well as with the host and its environment. Gut microbiota research is critical for gaining a thorough understanding of the relationships between gut microbiota and their hosts (Yukgehnaish et al., 2020). There is mounting evidence that bacteria interact with the hosts’ endocrine systems, giving them the ability to impact or be influenced by the wide range of physiological pathways that regulate the endocrine system (Garcia-Reyero et al., 2020). Previous studies have focused on salinity (Lozupone and Knight, 2007), pH (Fierer and Jackson, 2006); (Chu et al., 2013), and ecological interactions (Steele et al., 2011) as major determinants of free-living community composition. Microbial community variances have been linked to differences in hormone metabolism (Ridlon et al., 2013; Kwa et al., 2016), circulating hormone levels (Miller et al., 2017; Antwis et al., 2019), behaviour (Dinan et al., 2018), and even distorted gene expression in endocrine tissues (Martin et al., 2019). Recent evidence suggests that microbiota, particularly gut microbiota, can influence many physiological functions (Clemente et al., 2012) by establishing communication between the gut and proper brain functioning.

Microbial endocrinology has recently been recognized as an interdisciplinary field of study that connects microbiology, endocrinology, and neurophysiology. Its primary goal is to provide a paradigm for understanding the biological interaction between microorganisms and their hosts. The discovery of inter-kingdom signaling, which includes hormonal communication between microorganisms and their hosts, results in crosstalk between microbes and the endocrine system (Hughes and Sperandio, 2008). The direct action of microbes on gut mucosa and the enteric nervous system (ENS) can increase the microbiome’s output, allowing it to reach beyond the local GI compartment. In many ways, the gut microbiota resembles an endocrine organ due to its ability to influence the function of distal organs and systems (Forsythe et al., 2010; Evans et al., 2013). Although the processes driving gut-brain connections remain unknown, the gut microbiota has a substantial impact on the central nervous system and the idea to understand interaction of the gut-brain axis is becoming more crucial (Wang et al., 2019). Furthermore, the microbiota can alter the function of a variety of neurotransmitters and neuropeptides in the central nervous system, causing behavioural and physiological changes.

## Microbial Interactions Within Fish

A fascinating translational area of fish physiological study is the relationship between gut bacteria and host physiology. Like mammals, zebrafish have innate and adaptive immune systems for modulating interactions with microorganisms (Borrelli et al., 2016). Zebrafish research has shown that this fish is an ideal vertebrate developmental model for understanding the link between host-microbiota and host-pathogen, including the ontogenesis of gut microbiota. Microbiota can influence parasite colonization, replication, and virulence, implying that parasite-microbiota interaction is bidirectional. Colonization of the gut with certain microorganisms endows the host with a range of functions, including metabolism, nutrient absorption, immunological response, intestinal maturation, as well as regulates the expression of multiple cellular genes. Parasitic load can influenced by gut microbiota and that may lead to alteration in physiology and reproduction in fish. Similar findings have been obtained using (Rawls, 2007; Kanther and Rawls, 2010) fish models where organisms demonstrated to regulate metabolism. Gut colonizing microorganisms have a diverse interaction spectrum that can range from parasitism to mutualism depending on the physiological situation of the host (Méthot and Alizon, 2014). In some cases, interaction of microbes with host are asymptomatic, as many parasites caused disease on a random basis (Wammes et al., 2014). The interaction between eukaryotic parasites (helminths, protozoa, and fungi) and bacteria can alter the immune background of the gut interactions, ultimately affecting the host’s overall health status, either driving or protecting against dysbiosis and inflammatory diseases (Giacomin et al., 2015; Gause and Maizels, 2016). As a result, there are significant evidences that interactions between gut microbiome and parasites can impact each other’s pathogenicity, which is a key concern in aquaculture.

In herbivorous fishes (Nayak, 2010; Wu and Liu, 2012) microbial members aid in the digestion of cellulose. They also help the host-immune system for protection against pathogenic invaders in a better way and also influence innate immune responses. Boutin et al. (2014) provided the first evidence that host genotype regulates microbiota taxonomic diversity in brook char (*Salvelinus fontinalis*) and that particular host genomic areas regulate the acquisition of three specific bacterial genera (Lysobacter, Rheinheimera, and Methylobacterium) with antimicrobial activities.

The gut microbiota serves vital functions in the body, and abnormalities (dysbiosis) in its composition and diversity can reduce intestinal barrier protection and promote infectious pathogens (Infante-Villamil et al., 2021). However, any dysbiosis in microbiota can regulate peripheral and CNS function, altering brain transmission and host behavior (Collins et al., 2013). Probiotics, which have been widely marketed and consumed, primarily as dietary supplements or functional foods (Kumar et al., 2006, 2015), may benefit fish health by immunity enhancement (Kumar et al., 2008), and building a stable and robust intestinal ecosystem (Messaoudi et al., 2011). Probiotics fight for adhesion sites with pathogenic organisms, and can influence various activity in the gut, including GI tract function, gut immune function, cytokine production in mucosal cells (Lyte, 2010a; Llewellyn et al., 2016).

## The Diversification of Gut Microbiome: A Complex Endocrine Organ

**FIGURE 1:**
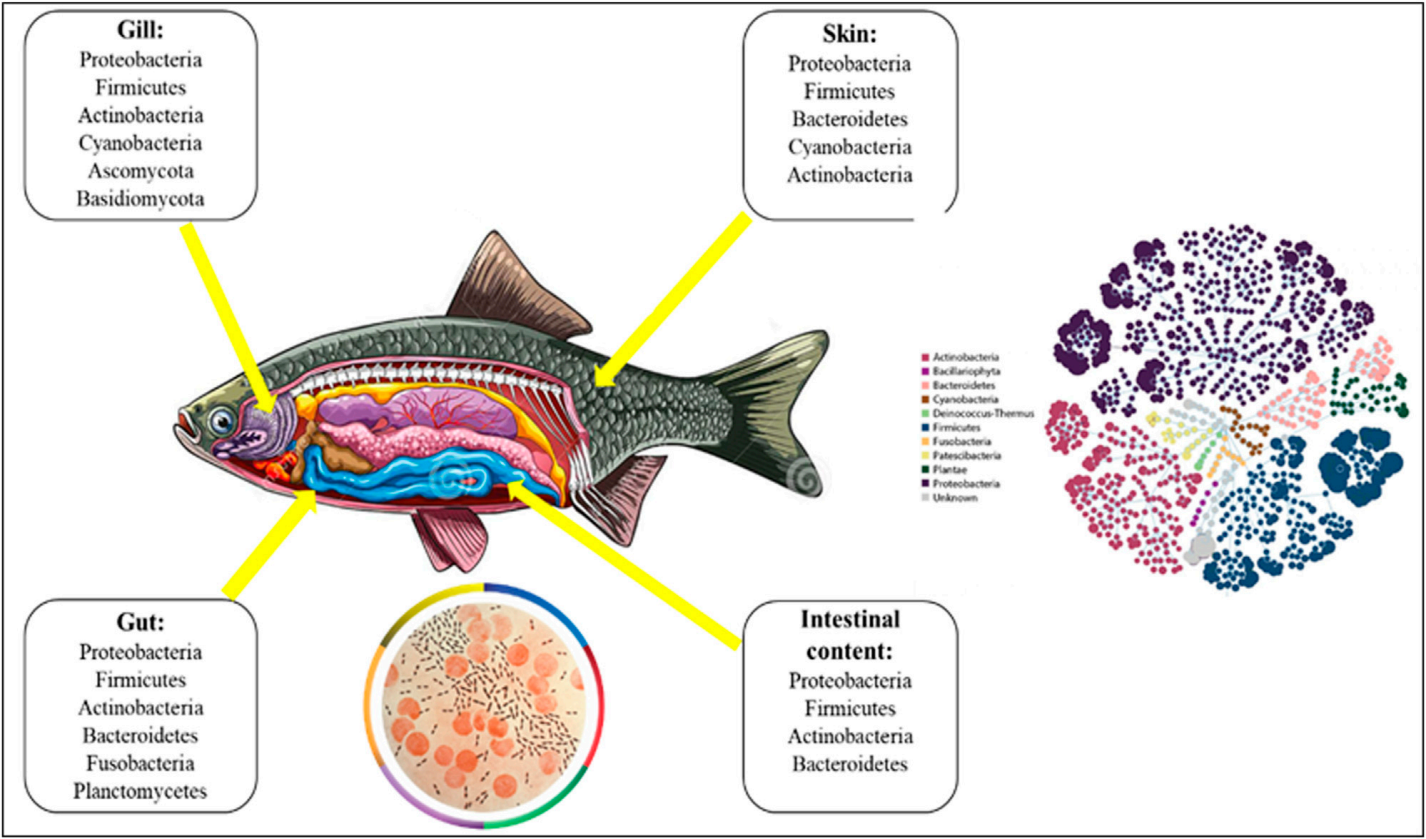
Composition of microbiota in fish.

**FIGURE 2:**
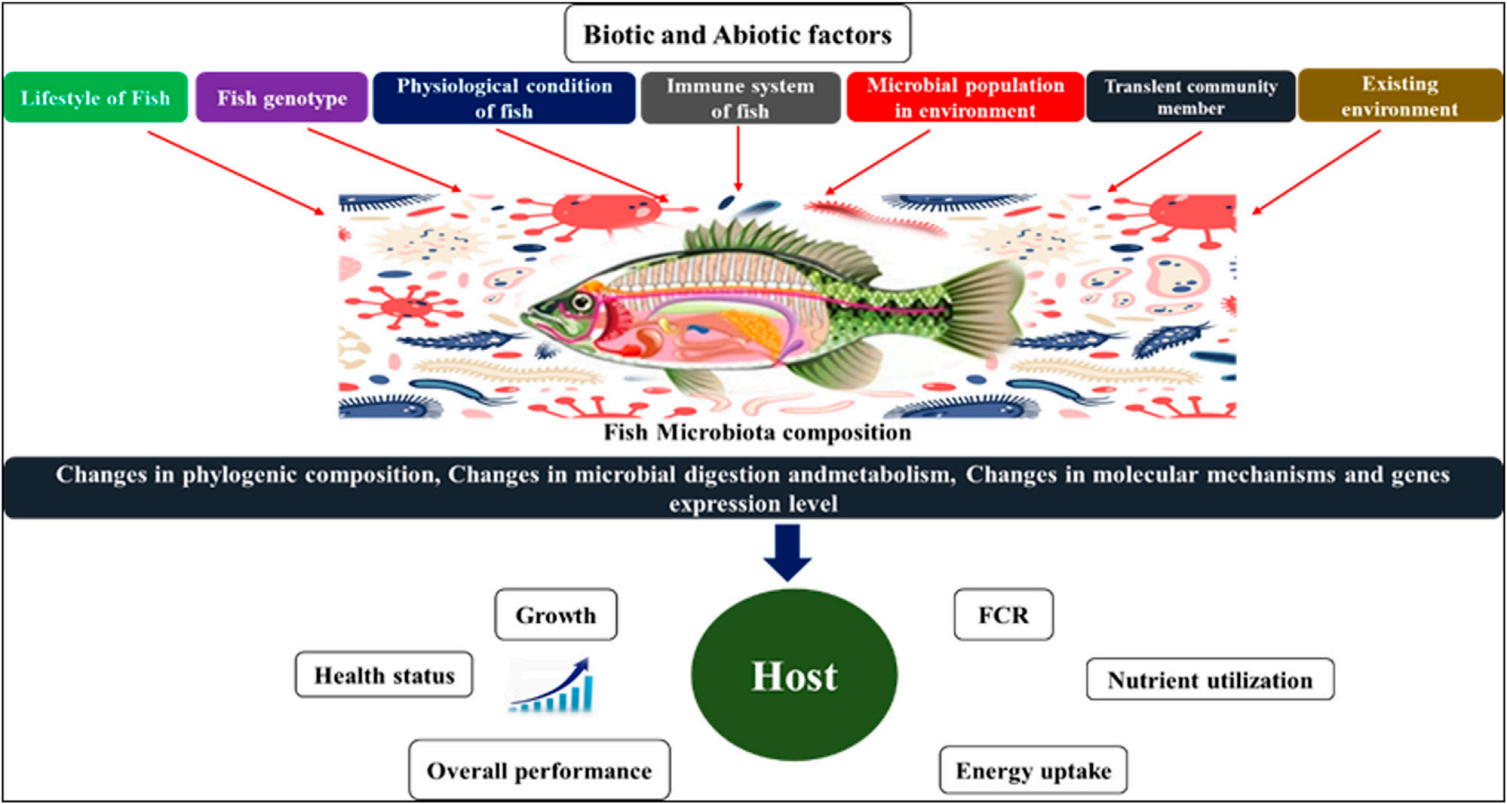
Factors that affecting the microbiome composition in fish and its impact on host physiology.

In contrast to other endocrine systems or organs that secrete only one or a few humoral agents, the gut microbiota has the capability to produce hundreds of products. It is much larger and biochemically more heterogeneous in terms of morphology and biochemistry. The biochemical complexity of the gut microbes even outnumbers that of the brain, and several of the microbiota producing hormones also function as neurotransmitters in the central nervous system (CNS). For example, several lactobacilli produce gamma-aminobutyric acid (GABA), the most important inhibitory neurotransmitter in the brain (Barrett et al., 2012), whereas monoamines such as noradrenaline, dopamine, and serotonin are also produced by certain strains of bacteria (Lyte, 2013). A dysfunctional reciprocal gut-brain relationship can lead to a range of illnesses, including inflammatory problems, inappropriate stress responses, changed behaviour and metabolic changes in fish. However, the processes behind these abnormalities in fish remain unknown. Microbial endocrinology is the study of microbes interacting with host neurophysiology. It has been extensively studied in the gut (Lustri et al., 2017) because it not only contains the majority of the animal body’s microbiota but also expresses a wide range of neuropeptides (Furness, 2016). Furthermore, the food contains neuroendocrine factors and precursors that can directly influence the intestinal microbiota (Lyte et al., 2019). In terms of mass and diversity, the skin is home to the body’s second most abundant microbiome (Grice and Segre, 2012). The gut and skin are major neuroendocrine organs (Slominski et al., 2000; Roosterman et al., 2006) that are innervated by a dense network of nerve fibers and are constantly in contact with the environment (Slominski et al., 2012). Large numbers of microorganisms colonize the epithelial surfaces of fish and all other vertebrates, forming commensal or mutual relationships with their hosts (Spor et al., 2011). The host microbiota system refers to the microbial communities, which include bacteria, archaea, eukaryotes, and viruses, that colonize various body surfaces such as the skin, gills, and intestine (Figure 1). Members of this community interact extensively with one another as well as their hosts, playing a significant role in modulating host physiology and homeostasis (Llewellyn et al., 2016). Davis et al. (2016) revealed a clear and significant link between the host and microbiota in zebrafish metabolism, immune system, and brain development as well as a variety of behaviour via the vagus nerve. The colonization of the gut in fish begins when larvae open their mouths and acquire bacteria from the chorion, feed, and water (Cicala et al., 2020). Before hatching the digestive tract, aquatic oviparous species’ eggs are surrounded by aquatic microbial communities. On the other hand, viviparous species are first exposed to the maternal environment before coming in contact with the ambient water (Longo and Bernardi, 2015). Along the microbiota ontogeny, biotic factors such as host genotype (Boutin et al., 2014), life stage cycle (Llewellyn et al., 2016), and population density (Dehler et al., 2017), as well as abiotic elements such as water chemistry, temperature, nutrition, and xenobiotics such as antibiotics (Sylvain et al., 2019), shape the host microbiota. These biotic and abiotic components are responsible for many physio-biological mechanisms in fish, as shown in Figure 2. According to (Nayak, 2010), the most frequently documented phyla in the salmonid gut microbiota are proteobacteria and firmicutes, suggesting that members of these bacterial taxa are especially well adapted to conditions in the fish intestine or their surrounding aquatic environment. Furthermore, *Pseudomonas* sp. can account for more than 60% of the population in salmonid gut microbial composition (Navarrete et al., 2008). *Clostridium* and *Aeromonas* are two significant groups that have been found in the intestines of rainbow trout (Pond et al., 2006). The digestive system of fish is also an environment for bacteriophages, and particularly bacterial composition, may be affected by bacteriophages (Bastías et al., 2010). The phyla Ascomycota’s Saccharomycetaceae, which includes the genus *Rhodotorula*, is commonly detected in the microbiota of both marine and freshwater fish (Gatesoupe, 2007), and it has also been discovered that *Candida* spp.*, Saccharomyces cerevisiae*, and *Leucosporidium* sp. have been reported in the rainbow trout intestine.

## Candidate Hormones of the Gut Microbiota

**FIGURE 3:**
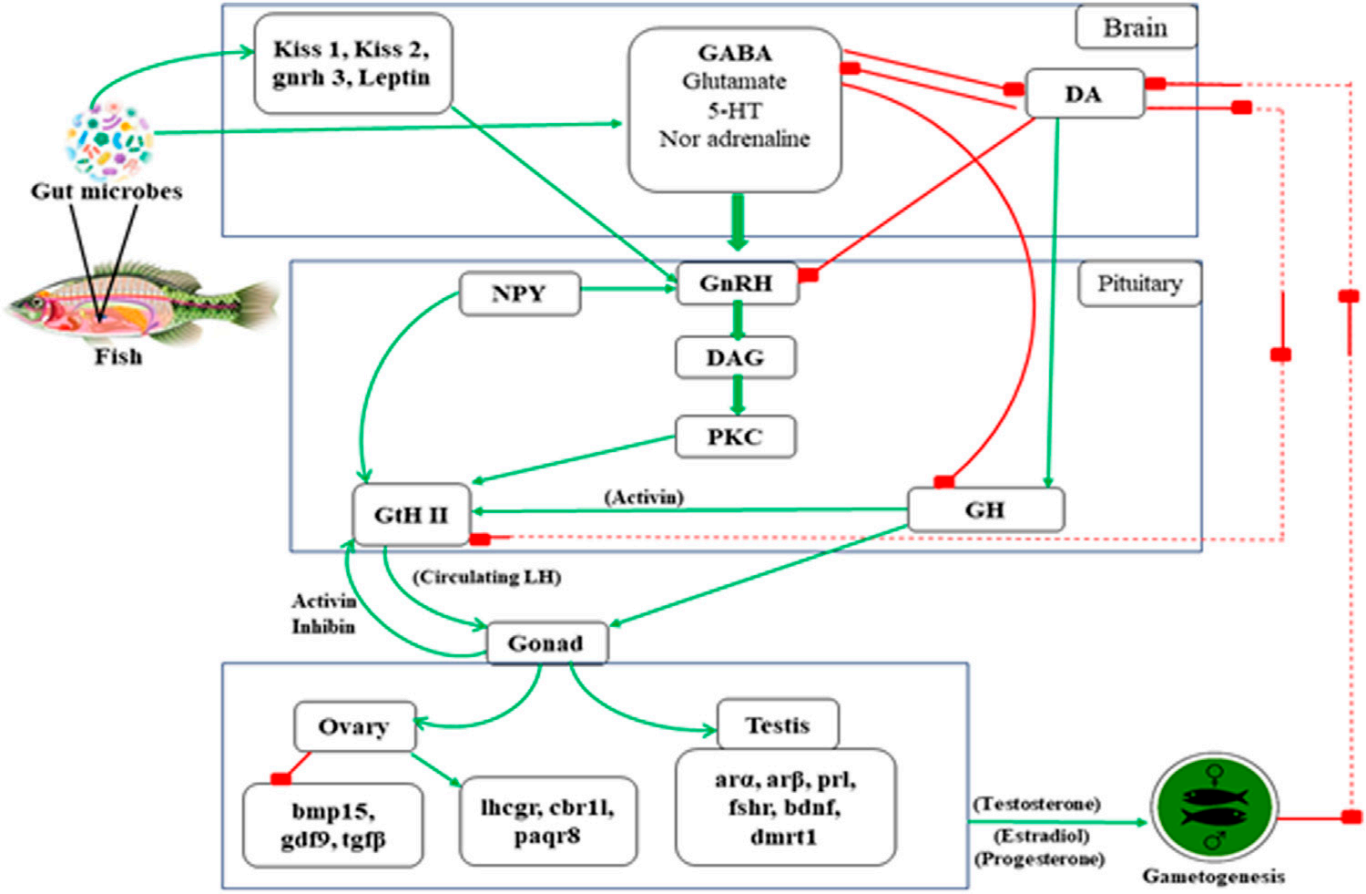
The possible mechanism involved in teleost reproduction induced by gut microbiota. * Arrow line indicates “positive feedback” and Box tipped arrow line indicates “negative feedback” in the above mentioned reproductive mechanism in teleost. **Text abbreviation–Kiss 1 (kisspeptin 1), Kiss 2 (kisspeptin 2), gnrh 3 (gonadotropin releasing hormone 3 gene), leptin, GABA (gamma-aminobutyric acid), 5-HT (5- hydroxytryptamine), DA (dopamine), NPY (neuropeptide-Y), GnRH (gonadotropin releasing hormone), DAG (diacylglycerol), PKC (protein kinase C), GtH II (gonadotropin II), GH (growth hormone), bmp15 (Bone Morphogenetic Protein 15), gdf9 (Growth differentiation factor-9), tgfβ (Transforming growth factor beta), lhcgr (Lutropin-choriogonadotropic hormone receptor), cbr11 (Carbonyl reductase 1-like), paqr8 (progestin and adipoQ receptor), arα (androgen receptors α), arβ (androgen receptors β), prl (prolactin), fshr (follicle stimulating hormone receptor), bdnf (brain-derived neurotrophic factor), dmrt1 (doublesex and mab-3 related transcription factor 1).

The gut microbiota plays an important role in the diversified physiology of fishes. The microbiota is known to mediate these physiological processes through mechanisms such as breaking down food components and strengthening the immune system by destroying toxins (Elahi et al., 2013). Modulation of hormone secretion revealed the nature of the host-bacteria relationship, which plays a vital part in the maintenance of the endocrine system. (Clarke et al., 2014). As previously discussed, the connection between host and bacteria is bidirectional as the microbiota has been proven to be affected by and affect the hormones of the host. After discovering that stress-induced neuroendocrine hormones can influence bacterial growth, Lyte and Ernst 1992 were the first researchers to develop the topic of microbial endocrinology research. Various studies on microbial endocrinology of mammalian models showed that hormone receptors in microbes are hypothesized to be a method of intracellular communication (Lyte, 1993). However, there is no clear understanding of the concept of microbial endocrinology in fish/teleost. A fascinating study discovered that many enzymes involved in host hormone biosynthesis (such as epinephrine, norepinephrine, dopamine, serotonin, melatonin, and others) may have evolved by horizontal gene transfer from bacteria (Iyer et al., 2005). Short chain fatty acids (SCFAs) are the primary byproducts of bacterial fermentation of carbohydrates and proteins in the intestine (Kovatcheva-Datchary and Arora, 2013). In many respects, they are the microbiota’s characteristic hormones, and may mediate many of the functions assigned to the microbiota *via* classical endocrine signaling. SCFA receptors and transporters are expressed throughout the GI tract and are significant for its functioning (Ganapathy et al., 2013). For example, SCFAs may influence enteroendocrine serotonin (5-HT) production as well as peptide YY (PYY) release, a key neuropeptide at various levels of the gut-brain axis (Holzer et al., 2012). The process of feeding behaviour and metabolism involving the brain and gut microbes is depicts in Figure 3. In this review, we screened the studies demonstrating multiple approaches to how gut microbiota impacts brain function and behavior in fish.

### Catecholamines

Adrenalin and noradrenaline were the first neurohormones which were shown to have antibacterial activity and this was correlated to be the origin of the concept of microbial endocrinology (Lyte, 2010a). Since the skin includes a large number of sympathetic nerve terminals, catecholamines are the primary autonomic skin neurotransmitters (Donadio et al., 2006). Many bacterial species have been defined for their response to catecholamines (Sarkodie et al., 2019), but the impact of these neurohormones on bacteria isolated from human skin has seldom been studied, and there is not a lot of relevant data available from an aquaculture standpoint. Catecholamines, like all other cutaneous factors discussed here, have no effect on the growth of these microorganisms. However, various bacterial catecholamine sensors have been found. The main one, discovered in *Escherichia coli*, is the QseBC system (Karavolos et al., 2013), however other receptors such as QseEF, BasRS, and CpxAR have also been identified.

### Pheromones and Sex Hormones

Pheromones are hormones that play key roles in sexual recognition, mating, aggressive behaviour, and dominance in aquatic animals such as fish. Pheromones are also known as ectohormones, which are chemicals secreted outside of one’s body that influence the behaviour of others. The influence of sex hormones on microorganisms have been studied for nearly 4 decades. *Prevotella intermedius*, for example, absorbs oestrogen and progesterone, which elevate its growth (Kornman and Loesche, 1982). Alteration in the expression of the oestrogen receptor (ER- β) can influence the diversity of the gut microbiota (Menon et al., 2020). This interaction is bidirectional, as some microorganisms have been implicated in steroid production or modification. *Clostridium scindens* is a bacterium that transforms glucocorticoids into androgens, which are male steroid hormones (Ridlon et al., 2013). According to (Adlercreutz et al., 1984), antibiotic use lowers estrogen levels, implying that gut bacteria play a role in oestrogen metabolism.

### Cutaneous Hormones

The skin has a plethora of neuropeptides, the vast majority of which have yet to be studied for their potential interaction with the cutaneous microbiota. This is the case with MSH and proopiomelanocortin (POMC) related peptides, which are generated in the *epidermis* and have antibacterial activity (Catania et al., 2006), but have received little attention in the context of skin physiology. At high non-physiological concentrations, other skin neuropeptides, such as neuropeptide-Y (NPY), vasoactive intestinal polypeptide, and galanin-related peptides, demonstrate antibacterial activity against microorganisms, including members of the cutaneous microbiota such as *Staphylococcus. Aureus*, *Streptococcus mutans*, and *Candida sp* (El Karim et al., 2008; Holub et al., 2011). The release of serotonin and melatonin by common cutaneous bacteria, as well as the presence of serotonin and melatonin dependent regulatory functions in the skin microbiota, have never been studied. Particular emphasis should be directed to γ-aminobutyric acid (GABA), which is generated by fibroblasts and immune cells in the skin (Ito et al., 2007) and released by interneurons implicated in itch transmission (Nigam et al., 2010). The effect of GABA on cutaneous bacteria was not particularly studied, however investigations were conducted on *Pseudomonas fluorescence*, one of the uncommon species of commensal skin proteobacteria, and *Pseudomonas aeruginosa*, which was primarily discovered in the skin under pathogenic conditions. Glutamate, another neurotransmitter released by primary sensory neurons in the skin (Miller et al., 2011), is also generated by several microbes, including corynebacteria (Persicke et al., 2015). Glutamate, as shown in the gut, may contribute to bidirectional communication between the cutaneous microbiota and skin (Baj et al., 2019).

### Hormones of Immunity and Stress

The intestinal microbiota influences the development and differentiation of the immune system. *Escherichia* spp. Produces dopamine, which is required for optimal brain function as well as the substrate for the production of the stress hormone norepinephrine (Tsavkelova et al., 2006). The gut microbiota can provide signals that stimulate the immune system’s normal development and immune cell maturation (Louis et al., 2014). There are numerous correlations that suggest, microbiome and hormones may influence the immune system via a common mechanism. The HPA axis monitors and integrates gut functions while also connecting the brain’s emotional and cognitive centres to peripheral intestine functions and mechanisms such as immune activation, enteric reflex, intestinal permeability, and entero-endocrine signalling *via* the enteric nervous system (ENS). An imbalance in the composition and diversity of the host’s microbiota might reduce intestinal barrier protection and favour infectious bacteria. Any dysbiosis in the host’s microbiota can regulate peripheral and CNS function, altering brain signals and behavior (Collins et al., 2013). In this context, two specific species, *L. helveticus* and *B. longum*, have been found to reduce stress hormone cortisol levels as well as anxiety-like behaviour in both rats and healthy humans (Messaoudi et al., 2011), suggesting its prominent role in downregulation of cortisol, steering the peripheral and CNS function towards signaling restoration of homeostasis. Zebrafish as a model illustrate the function of the gut microbiota in maintaining equilibrium in the gut-brain axis via immunomodulation, protection, nutrition and metabolism, illness, as well as directly add to an anxiety like phenotype (Mohanta et al., 2020). Manipulation of the zebrafish gut microbiota resulted in greater resistance to infections, stimulation of the immune response, growth enhancement and improved gut physiological status. The same collection of hormones and receptors exist in both the immunological and neuroendocrine systems. Glucocorticoids, such as corticosterone and cortisol, regulate inflammation and have an impact on both innate and adaptive immunological responses in fish (Franchimont, 2004).

### Neurohormones

Asano et al. (2012) were the first to establish that the microbiota was capable of producing biologically active neuroendocrine hormones *in situ*. The GIT is the vertebrate body’s largest endocrine organ (Holst et al., 1996). A wide range of hormones and signaling molecules are secreted by various types of endocrine cells along the length of a fish’s GIT (Holmgren et al., 1986; Abad et al., 1987). Neurohormones are hormones that are released by neuroendocrine cells in response to neurological input. They can function as neurotransmitters despite being discharged into the bloodstream to have a systemic effect. Microbiota are considered to influence behaviour (such as anxiety in animals) through neurohormone precursors (e.g., serotonin, dopamine) (Lyte, 2013). Neurohormones such as serotonin, dopamine, acetylcholine, and norepinephrine can be produced and responded to by gut microbes (Roshchina, 2010). The fish host has a large number of neurochemicals. Certain bacteria in the gastrointestinal system, for example, produce large amounts of γ-aminobutyric acid (GABA), the primary inhibitory neurotransmitter found in the mammalian brain (Obata et al., 2013) as well as immunomodulatory properties (Bjurstöm et al., 2008). The metabolic pathways employed by the microbiome to manufacture these neurochemicals are similar to those found in the host, which is critical in recognizing the ubiquitous nature of neurotransmitters produced by members of the microbiota and their relationship to the host. GABA is synthesized by both eukaryotic and prokaryotic organisms, and the essential enzyme, glutamate decarboxylase, has been identified in a variety of Gram-positive and Gram-negative bacteria, including *Staphylococcus*, *Bacillus*, *Streptococcus*, and *Pseudomonas* (Hammer et al., 2019). Even common bacterial Quorum sensing (QS) components have the potential to act as neurotransmitters. This is especially true for N-(3-oxododecanoyl)-L-homoserine lactone, which is generated by Gram-negative bacteria (Hughes and Sperandio, 2008). Critical reviews of the scientific literature reveal numerous reports claiming, microorganisms ability to produce and respond to neuroendocrine hormones has potent physiological consequences for the host, providing solid evidence about the intersection of the fields of microbiology and neurophysiology. Knowing the microbiota composition in the host is critical for identifying processes by which the microbiome regulates host neurophysiology and ultimately behaviour. The ability of microorganisms to create neuroactive components is dependent on the availability of appropriate substrates, which has yet to be adequately addressed. As a result, the significance of nutrition is critical in assessing the microbiome’s ability to create neuroactive chemicals. The microbes produce a variety of neuroactive substances like catecholamines, histamine, and other compounds that can stimulate the host’s neurophysiology, either directly through interaction with receptors present in the GI tract or via passive diffusion through the gut wall and finally enters the portal circulation. *Escherichia* spp. produces dopamine, which is essential for optimal brain functioning as well as the substrate for the formation of the stress hormone norepinephrine. The evolutionary pathway of intercellular signaling shares this level of communication and its mediation. It has been postulated that these pathways evolved first in bacteria and were then adopted by eukaryotic cell systems via late horizontal gene transfer.

## The Microbiota-Gut-Brain Axis (Gut Hormone- Brain Crosstalk)

**FIGURE 4:**
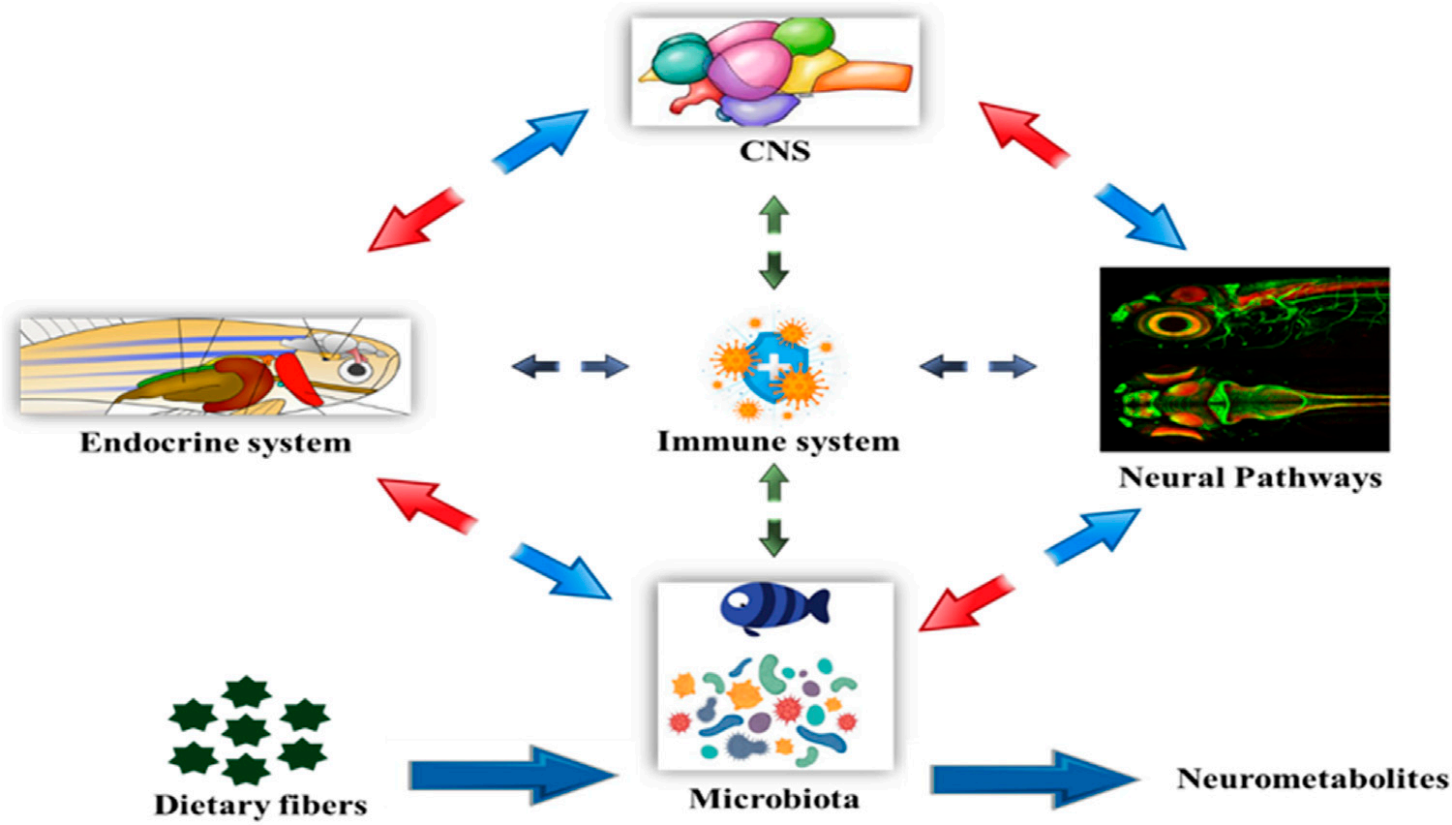
Bi-directional relationship between the gut microbiota and brain function. The bacterial metabolites of dietary fibers like SCFAs are neuroactive compounds that activates neural circuit, endocrine system, mucosal immune responses, CNS function and modulating signaling pathways influencing the host behavior.

Entero-endocrine cells (EECs), are considered as one of the largest endocrine systems that control food uptake and energy homeostasis in the body, can be elevated by gut bacteria to release peptides. EECs are synaptically coupled to vagal afferent terminal postganglionic sympathetic nerves and facilitate bidirectional communication between the ENS, CNS, and gut, producing peptides such as galanin, orexin, leptin, and gastrin (Forsythe and Kunze, 2013). The gut is a highly innervated organ with its own neural system called the enteric nervous system, which is constantly in interaction with the central nervous system (CNS) via nerves like the vagus. The CNS influences intestine function *via* the hypothalamic-pituitary-adrenal (HPA) axis, as well as the sympathetic and parasympathetic autonomic nerve systems (ANS). Furthermore, the ANS is another avenue *via* which the CNS impacts the intestinal microbiota. The CNS receives neurological and chemical signals from the gut on a continuous basis and is in charge of integrating this information and creating appropriate responses to maintain homeostasis. According to current data, the gut influences CNS activities primarily via the immune system, neuroimmune mechanisms, neurotransmitters, and ANS, which typically involves the vagus nerve, enteric nervous system, enteroendocrine signaling, and metabolites derived from the gut microbiome (Cryan et al., 2018). To influence the connection between gut and brain, neural networks act in both ascending (gut-to-brain) and descending (brain-to-gut) directions (Sternberg, 2006). This communication system between the gut and the brain is made up of complicated loops of neurological responses (Genton and Kudsk, 2003). The gut microbiota influences the brain through a wide variety of compounds, including neurotransmitter homologs and other metabolites. On one hand, these substances are recognized by host cell receptors and activate nerve endings, immune cells, or EECs, a process known as the microbiota-gut-brain axis. Some molecules, on the other hand, can permeate the intestinal barrier, enter the circulation, cross the blood-brain barrier, and deliver to the brain, a process known as the gut microbiota-brain axis Figure 4. The gut’s innervation, like its microbiome, is not uniform along its length. As a result, it is critical to understand how one microbial species that produces a neuroactive chemical might have an influence on behaviour in one section of the gut but not another. The gut microbiota is established early in infancy, but it can be affected later by a variety of conditions that affect its development and diversity. Microglia dysfunctions and deficits have been identified in a variety of brain areas, including the cortex, corpus callosum, hippocampus, olfactory bulb, and cerebellum (Erny et al., 2013). These findings are consistent with a growing body of research on microbiome-neuro-immune interactions that influence behavioural and physiological abnormalities in mice models of multiple sclerosis, depression, stroke, and other diseases. The gut microbiota regulates homeostasis, which includes brain development, and has a significant impact on brain function. The microbiota is required for the normal development of the mucosal and systemic immune systems, as well as nutrition absorption and metabolism. The microbiome impacts the hypothalamic-pituitary-adrenal axis (HPA), the stress response, and behaviour, particularly anxiety-like and locomotor behaviour, which may affect the host’s food behaviour and energy homeostasis. The microbiota can also affect the function of the HPA axis by replenishing corticotrophin releasing hormone (CRH) concentrations and regulating excessive corticosterone levels (Carnevali et al., 2013). The complete microbiota-gut-brain axis (gut hormone-brain crosstalk) pathway is depicted in Figure 3. Currently, research is being conducted to determine the precise processes by which gut microbiota composition influences brain function in fish models. The precise mechanism of microbial endocrinology in fishes is unknown. Researchers are attempting to decipher the signaling systems that modify gastrointestinal function and host behaviour by interconnecting the gut-brain interface.

## Fish Microbiota and the Reproductive Axis

It is difficult to establish unambiguous, mechanistic linkages between microbiota, the endocrine system, and reproductive hormone control, and just a few studies have been conducted in this area. To date, the majority of research on the involvement of microbiota in reproduction has been descriptive in nature, evaluating changes in microbial communities inside specific physiological niches (i.e., male and female reproductive tracts) during the reproductive cycle (Moreno and Simon, 2019). *Lactobacilli* are the most common bacteria in the vaginal microbiome of animals (Moreno and Simon, 2019). These findings imply that hormones may influence vaginal microbial communities and/or vice versa. Furthermore, they demonstrate that, while microbiomes differ in terms of community composition, functional niches are usually conserved, allowing different microbial communities to perform identical functions across species. A recent human study discovered that men with good sperm quality (high motility and normal morphology) had an increased abundance of *Staphylococcus* spp. and *Lactobacillus* spp, respectively, and that the microbiomes of the male and female reproductive tracts are generally similar (Baud et al., 2019). Hormones are well known to play a role in sperm maturation inside the male reproductive tract (Miura et al., 1992) and the development of sperm motility (Tan et al., 2019) in comparative models, particularly fish. Gut microorganisms can influence reproductive endocrine control by directly modifying hormones, hence altering their bioavailability and efficacy (Kunc et al., 2016). Members of the gut microbiota frequently express a variety of hormone-converting enzymes, particularly conjugated steroids, such as glucosidases, glucoronidase, and hydroxysteroid dehydrogenases (Kunc et al., 2016; Kwa et al., 2016). Probiotic treatment of zebrafish (*Danio rerio*) with *Lactobacillus rhamnosus* resulted in increased ovarian function, which was associated with increased ovarian expression of genes positively associated with oocyte maturation and ovulation and downregulation of genes negatively associated with these processes (Carnevali et al., 2013). Gut bacteria have been demonstrated to enzymatically modify all steroid families (Kunc et al., 2016). However, it is exciting to speculate on how bacteria can contribute to these processes, either through the production of biomolecules with signaling potential or through other means. Investigating such connections could be a fruitful topic of future aquaculture research.

## Microbial Derived Metabolites and Neuroactive Substances

**FIGURE 5:**
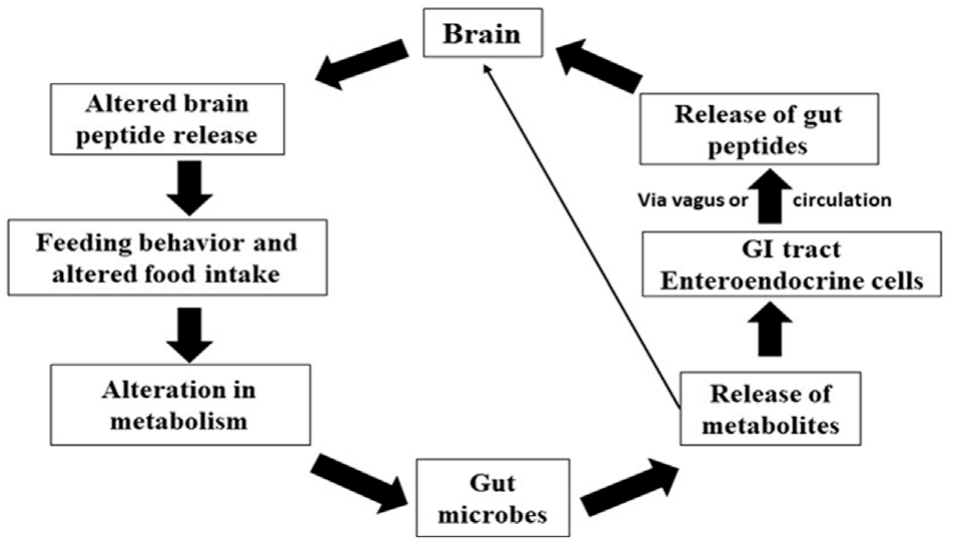
The process involved between brain and gut microbiota related to feeding behavior and metabolism.

The gut-brain transmission can change neurochemistry of brain such as gamma-aminobutyric acid (GABA), the principal inhibitory neurotransmitter in the CNS, and serotonin [5-hydroxytryptamine, (5-HT)] levels (Clarke et al., 2014). Neuroactive chemicals such as 5-HT, GABA, and tryptophan metabolites all play significant roles in CNS regulation (Clarke et al., 2013). The gut microbiota degrades nutrients, which are then digested by host cells and some of these metabolites are implicated in neurological activity. Gut bacteria in humans generate amino acids such as GABA and tryptophan and monoamines such as serotonin, histamine, and dopamine, which play essential roles in the brain as neurotransmitters or neurotransmitters precursors (Frank et al., 2008). These compounds can target the CNS via the circulation and impact neurons in the ENS (Semova et al., 2012; Brugman, 2016). The chemicals released by gut microbes in fish are intriguing for example, *Candida* and *Escherichia* can use tryptophan to make 5-HT, whereas *Bacillus* can produce dopamine. GABA has been produced by *Lactobacillus* spp. and *Bifidobacterium* spp, noradrenalin by *Escherichia* spp*., Bacillus* spp. and *Saccharomyces* spp*.,* serotonin by *Candida* spp, *Streptococcus* spp*., Escherichia* spp, dopamine by *Enterococcus* spp. (Semova et al., 2012). These bacteria are thought to influence the CNS through a particular mechanism by which they can create neurochemicals that are structurally identical to neurotransmitters produced by neuronal cells. Gut bacteria aid in the fermentation of starch or dietary fibres in the colon, resulting in the creation of SCFAs such as butyrate, acetate, and propionate (Sternberg, 2006). Butyrate is immediately used as an energy source by colonocytes. In contrast, propionate and acetate are transferred from the intestinal lumen into the host’s blood circulation and to the organs, where they act as substrates or signal molecules. Acetate and propionate aid in synthesizing ATP in the muscles and liver (Hernandez et al., 2019). SCFAs influence energy metabolism in the colon, food intake, and gut homeostasis by binding to their receptors, which include G protein-coupled receptors, immune cell activity, hormone generation, inflammation, and activation of host epithelial cell signaling pathways (Genton and Kudsk, 2003; Moloney et al., 2014). The vagus nerve, immunological activation, and the generation of microbial and neurometabolites such as short-chain fatty acids (SCFAs), vitamins, and neurotransmitters are all implicated in this bidirectional pathway. The fish gut microbiota regulates hormone-like compounds, and the various pathways for those actions are presented in Figure 5. The precise method by which they alter brain functions in aquatic animals is unknown yet to be unrevealed and it is hypothesized that, the neuroactive chemicals produced by the gut microbes can pass the gut mucosal layer and act on the enteric nervous system.

## Effect of Microbial Metabolites and Neuroactive Substances on Reproduction

Gonadal maturation, sex steroid, sex pheromone synthesis, and reproduction of fish are regulated by gonadotropin-releasing hormone (GnRH) and dopamine (DA) following positive and negative feedback, respectively (Omeljaniuk et al., 1987; Peter et al., 1988). The interaction between the neuropeptide GnRH, catecholamine, DA, and GABA plays a significant role in the fish reproductive cycle (Trudeau, 1997). Trudeau et al. (2000) has mentioned that the stimulatory actions of GABA are inhibited by DA, which also has a down regulatory action on GnRH receptors of goldfish pituitary (Leeuw et al., 1989). The positive feedback activities of sex steroids appear to result from increased pituitary sensitivity to GnRH and increased GABAergic activity rather than alterations in DA function (Trudeau et al., 1993). In the goldfish brain’s telencephalon preoptic–hypothalamic (TEL-POA-HYP) area, GABA increases LH release by activating GnRH and inhibiting DA neurons (Trudeau et al., 2000). It has been demonstrated by increasing GABA levels with the irreversible inhibitor of GABA-T vinyl gamma (GVG), intraventricular GABA injection, and intraperitoneal injections of GABA agonists. Thus, activation of endogenous GABAergic pathways activates pituitary secretion and transcription. As a result, to control LH release, the GABAergic system transduces both external environmental and internal hormonal feedback signals. Several 5HT receptor subtypes have recently been discovered to be involved in releasing GnRH and LH. *In vitro* activation of the ionotropic receptor, 5HT3A, enhanced LH beta mRNA expression in rat pituitary (Quirk and Siegel, 2005). This could be a way for the pituitary to respond quickly to environmental changes. Only a 5HT2-like receptor at the GnRH cell body or nerve terminal is known to have stimulatory effects on LH release in goldfish (Somoza and Peter, 1991), red seabream (Senthilkumaran et al., 2001) and Atlantic croaker (Khan and Thomas, 1992). In the hypothalamus of sexually mature rainbow trout, the 5HT2 antagonist ketanserin binding was higher than in juveniles, suggesting that 5HT2-type receptors are involved in reproduction (Agrawal and Omeljaniuk, 2000).

Furthermore, 5HT suppresses the release of GH in goldfish (Somoza and Peter, 1991). As a result, fluctuations in brain receptor levels may play a role in the seasonal cycle of reproduction and growth (Marchant and Peter, 1986; Tecott and Abdallah, 2003). The Gonadotropin-releasing hormone (GnRH) neurons are controlled by a signaling network that comes from the brain via afferent nerve. The neuropeptide (NPY), most abundant peptide in the neurological system is triggered by circulating leptin and insulin levels and has a direct influence on GnRH, follicle-stimulating hormone (FSH), and luteinizing hormone (LH) secretion (Won and Borski, 2013).

**FIGURE 6:**
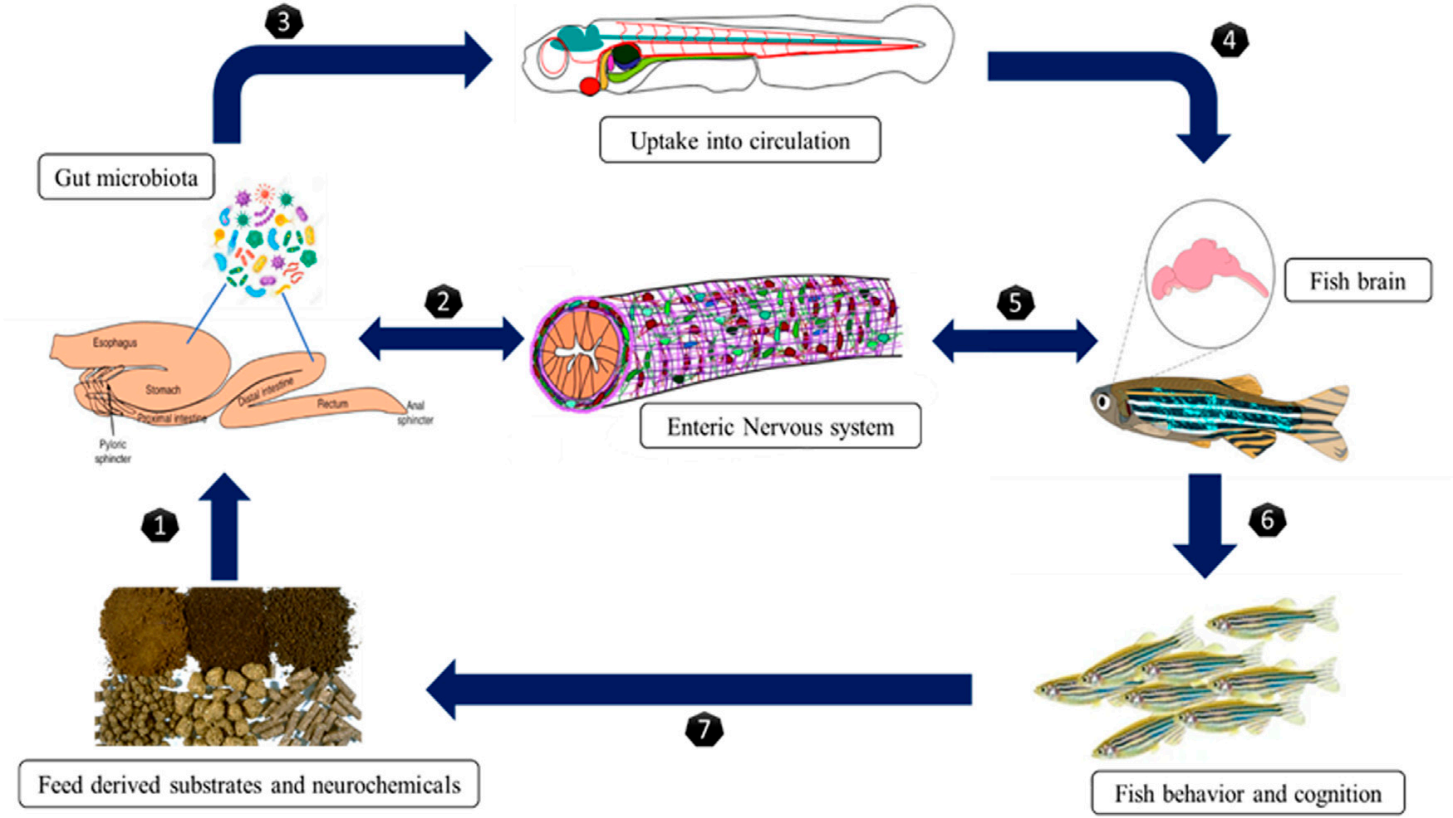
The microbiota-gut-brain axis (gut hormone-brain crosstalk).

In this review, we are bringing out the fact that the influence of microbes from the gut, skin, and other parts of the body, which help to regulate fish reproduction through the endocrine system. Transcriptomic and proteomic studies evidenced many neo secreted proteins by gut microbes such as *Lactobacillus*, and these effector molecules influence host physiology (Gioacchini, 2011; Semova et al., 2012; Carnevali et al., 2013). van de Wetering et al. (2002) have reported that intestinal bacteria from zebrafish influence the epithelial cells by enhancing β-catenin stability, promoting cell proliferation. Blache et al. (2004) described that β-catenin is controlled by Wnt signaling; its protein is translocated into the nucleus, interacting with proliferative target genes, such as c-myc and Sox9. Sox9 is also involved in chromosomal control of testis differentiation in teleost (Avella et al., 2012). Gioacchini (2011) studied that *Lactobacillus rhamnosus* induced a significant enhancement in Kiss1, Kiss2, and leptin in the brain, concomitant with an increase in gnrh3 gene expression. These genes act on the pituitary level, stimulate FSH and LH production (Zieba et al., 2005), and control the steroidogenesis process (Moschos et al., 2002). The microbial organism also affect the expression of cyp19a in the ovary, erα, and vitellogenin (vtg) in fish liver. Also, it increased the gonadosomatic index (GSI) and enhanced the vitellogenic follicles in the fish ovary (Gioacchini, 2011). As shown in Figure 6, an increase in transcription of genes coding for signals involved in the maturation phase, such as lhr, 20β-hsd, mprb, cyclin B, activinbA1, and smad2, was observed, along with downregulation of genes coding for local factors that prevent premature oocyte maturation, such as tgfβ, gdf9, and bmp15. In addition to estradiol (E2) several members of the transforming growth factor (tgf) superfamily may play a role in vitellogenesis regulation. During vitellogenesis, mRNA levels of the activin A subunit, also known as inhibin βA (inhba), rise potentially promoting follicle growth (Wang and Ge, 2003c; 2003a; 2003b). Tgfβ1 mRNA increases the expression of fshr, implying that vitellogenesis may be influenced (Kohli et al., 2005). Finally, it was demonstrated that bone morphogenetic protein 15 (bmp15) could inhibit precocious follicle maturation (Clelland et al., 2009). Higher levels of genes coding for signals inducing oocyte maturation (lhcgr, cbr1l, paqr8) were found in the ovaries of zebrafish treated with *L. rhamnosus* (Carnevali et al., 2013; Gioacchini et al., 2014); an opposite trend was associated with the transcription of local factors involved in preventing oocyte maturation (bmp15, gdf9, and tgfβ). Valcarce et al. (2015) observed *P. acidilactici* a significant increase in leptin, bdnf, and dmrt1 gene expression after a 10-day dietary administration of *Pediococcus acidilactici*. Higher sperm quality was associated with increased levels of spermatogenesis genes activin, arα, arβ, pr1, and fshr (Valcarce et al., 2015); the entire mechanism is depicted in Figure 6.

## Importance of Microbiota on Fish Physiology

### Nutrient Digestion and Metabolism

(Rawls et al., 2006) discovered that the GI microbiota can influence the expression of 212 genes in the zebrafish digestive tract, some of which are associated with epithelial proliferation stimulation and nutrition metabolism optimization. The zebrafish intestinal environment favours a specific group of bacteria that are regulated by host anatomy, physiology, nutrient availability, and immunology (Baumgart and Sandborn, 2012) and these microbes are involved in dietary functions. According to (Smriga et al., 2010), members of the Proteobacteria, Bacteroidetes, Firmicutes, and Fusobacteria phyla may aid in digestion in fish such as parrotfish, snapper, and sturgeons by offering a variety of enzymes. Fusobacteria, which have been found to colonise the stomach of zebrafish (Roeselers et al., 2011), can excrete butyrate (Kapatral et al., 2003) and produce vitamins (Roeselers et al., 2011), both of which may exerts a positive effect on fish health. *Cetobacterium somerae* has been found in a variety of fish species, including rainbow trout (Kim et al., 2007), common carp (Omar, 2012), zebra fish (Roeselers et al., 2011), and goldfish (Silva et al., 2011). As *Cetobacterium somerae* produces substantial amounts of vitamin B12 (cobalamin) in gut, therefore it has been proposed that this species serves as a source of cobalamin for some freshwater fish species (Tsuchiya et al., 2008). (Ray et al., 2012) reported that the fish gut microbiota may contribute to host nutrition by delivering enzymatic activities that have a positive effect on fish digestive processes. The ability of grass carp to digest plant stuff has long been linked to a greater abundance of cellulolytic bacteria in the gut of herbivorous fish (Li et al., 2009). Cellulolytic *Aeromonas* predominates in the gut bacterial community of grass carp, followed by *Enterobacter*, *Enterococcus*, *Citrobacter*, *Bacillus, Raoultella, Klebsiella, Hydrotalea, Pseudomonas*, and *Brevibacillus*, and a raise in plant-fiber consumption enhances the range of cellulolytic bacteria (Li et al., 2009). Cellulose degrading bacteria like *Clostridium, Aeromonas, Cellulomonas*, and *Bacteroides* along with other nitrogen fixing species are found to supply assimilable carbon to the wood eating fish *Panaque nigrolineatus* (Watts et al., 2013). Clostridia also dominate the microbial flora of the intestine in certain marine herbivorous fish species (Clements et al., 2007, 2014). In contrast to the cellulolytic function of the microbiome in herbivorous species, carnivorous species have significantly larger levels of lipase, protease, and trypsin producing bacteria and activity of trypsin (Li et al., 2009), confirming the significance of microbiota in host digestion. Lactic acid-producing bacteria (LAB) was found in greater abundance in Atlantic salmon fed a plant-based diet than in those on fishmeal-based diets, implying a potential role in digestion (Catalán et al., 2018).

### Immunity and its Ability to Withstand Stress

**FIGURE 7:**
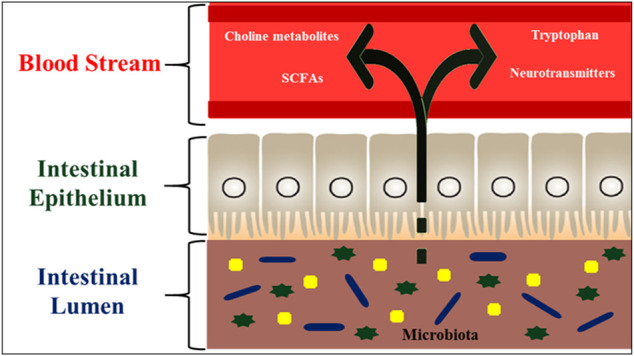
Hormone like metabolites regulated by the fish gut microbiota. These microbial metabolites such as SCFAs (having signaling functions) are secreted into the fish intestinal lumen, and transported to the effector organs, including the brain, via blood stream. The gut microbiota is also capable of producing or releasing neurotransmitters such as serotonin or regulating the availability of precursors such as tryptophan. The microbiota also regulates the bioavailability of choline and its metabolites.

Some studies on zebrafish and mice provide insights into the microbial-host molecular dialogues that affect numerous host functions, including feeding, immunology, and development (Rawls et al., 2006; Round and Mazmanian, 2009). Because the intestinal microbiome is required for nutritional metabolism, the composition of the zebrafish gut microbiome can have a significant impact on disease aetiology (Rawls, 2007). Apart from digestion, alterations in microbiome composition caused by environmental stress result in compromised immunity in the host (Dawood, 2021). Gut bacteria create significant amounts of short-chain fatty acids (SCFAs), which are absorbed in the intestine via simple diffusion or specialized receptors and provide resistance to harmful invaders (Montalban-Arques et al., 2015; Maji et al., 2018). As a result, the functional repertoire of gut microbiota appears to be synergistic with the needs of the host. The composition of gut microbiota may regulate CNS function *via* numerous communication channels such as neurological, hormonal, humoral, and immunological (activation of the mucosal immune system). The gut-to-brain microbial axis is regulated by stress factors and works through changes in intestinal motility and permeability as well as the liberated neurotransmitters and mucus. There is a bi-directional interaction that regulates gut-to-brain communication in both health and sickness, but little is known about how bacteria can influence this communication (Figure 7). There is a possibility that alterations in microbiota can affect CNS function, despite the fact that the composition of human intestinal microbiota changes over time, as food and overall health changes (Brugman, 2016). Zebrafish and mammals share a high degree of resemblance in the acquired immune system as well as in the digestive system and most interestingly, the intestinal tract harbours a diverse community of bacteria residing in humans and other terrestrial and aquatic animals, including fish.

## The Gastrointestinal Microbiome and its Bidirectional Regulations

Upon meal consumption, nutrients in the GI tract activate a variety of hormones, peptides, and neurotransmitters that are responsible for bidirectional transmission along the gut-brain axis (GBA). Enteroendocrine cells (EEC), which are specialized cells in the GI epithelium that excrete key signaling molecules and peptides, are responsible for much of this bidirectional communication (Sandhu et al., 2017). Cholecystokinin (CCK) and peptide YY (PYY) are two of these hormones that signal satiation via direct EEC-nerve transmission or indirect paracrine pathways (Sandhu et al., 2017; Butt and Volkoff, 2019). SCFAs have been shown to regulate inflammatory responses and metabolism, but they also influence neuroendocrine hormone release via interactions with EEC surface receptors (Cani et al., 2013). The gut microbiome can also influence bile acid synthesis and secondary bile acid formation (Haygood and Jha, 2018)**,** both of which influence EEC neuropeptide release via interaction with the apical bile acid GPCR, TGR5, and the farnesoid X receptor (FXR), a nuclear receptor responsible for maintaining glucose tolerance and insulin sensitivity (Cani et al., 2013; Sandhu et al., 2017). While the majority of studies on the processes underlying microbial modulation of GBA signaling have been conducted on human, mouse, and rat models, evidence suggests that the microbiome’s influence on neuroendocrine signaling is conserved across many animal taxa. Few studies in fish have looked at the direct mechanism of microbiota-gut-brain axis transmission, but zebrafish (*Danio rerio*) studies have shown that microbial colonization is required for appropriate epithelial fatty acid absorption, as well as lipid accumulation and metabolism (Sheng et al., 2018).

## Temperature Rise and its Impact on Gut Microbes

Animals are hosts to a wide range of bacteria, fungi, protists, and viruses (Baquero et al., 2013). Climate change/temperature rise can have wide impacts on animal health. Climate change affects animals and can be viewed as influences on a host-microbiome assemblage, or “holobiont,” given the co-dependence of hosts and their resident microorganisms (Roughgarden et al., 2018). To understand the pattern of change in fish gut composition as a result of climate change, it is necessary to assess the effects of climate change/temperature rise on fish and their microbial symbionts. Microbiologists may now use genetic techniques to investigate microbial life on all surfaces, even on and within live humans, due to advancements in DNA sequencing technologies (Caporaso et al., 2012). Changes in the environment, stress, nutrition can influence host microbiomes, which ultimately affect fish health. Increase in temperature is also expected to have an impact on microorganisms, as changes in temperature can affect ambient microbial reservoirs, and rising temperatures may have an indirect effect on animal microbiomes as well. While an animal may have the ability to adapt to environmental changes, the loss of essential microorganisms may impair its health and ability to live (Heiman and Greenway, 2016). Climate change/temperature rise influences the physical and chemical properties of aquatic habitats, which in turn influence organism physiology and phenology, and, ultimately, the composition of food webs. Fish live in water, and as a result, changes in aquatic ecosystems have an immediate impact on them. They are poikilothermic; changes in external temperature have a direct effect on their body temperature. Depending on the species and their spawning window, seasonal temperature changes have either accelerated or slowed the spawning process. Temperature and rainfall in tropical and subtropical climates can stimulate long-term reproductive activity. Atypical temperature regimes can influence the length and timing of the reproductive season, as well as the quantity and quality of reproductive output (Durant et al., 2007). As a result, temperature can have a variety of effects on the neuroendocrine reproductive axis (Pankhurst, 2011). Warmer water, for example, can influence GnRH secretion, clearance, and gonadal steroidogenesis in salmonids (Wang et al., 2009; Pankhurst, 2011). Rising temperatures may have an immediate negative impact on animal fitness due to effects on physiology, but they may also have an indirect impact by disrupting mutualisms between animals and other creatures. The effects of temperature on species relationships have been widely studied in symbiosis between eukaryotes. Some of the most common symbioses that animals enter are those with bacteria and archaea (McFall-Ngai et al., 2013), underlining the necessity of understanding the impact of temperature on interactions with these species as well. Microbial communities reside both within and outside the body of an animal. Although some of these animal-associated microbial communities are sparse, unstable, or have little functional value for their hosts (Hammer et al., 2019), many play an important role in host phenotypes and fitness. As global temperature regimes change, any impact on the composition of animal gut microbial communities may affect their activities, with repercussions for host phenotypes and fitness. Understanding how ambient temperature affects animal gut microbiota may thus aid in predicting future reactions of animal lineages and communities to climatic change. Temperature increases, in particular, have been linked to changes in community membership and relative abundances of certain bacteria (beta diversity) in host humans. Data suggests that each host species exhibits a distinct microbial response to thermal stress, but some gut bacterial taxa, particularly Firmicutes and Proteobacterial lineages, exhibit consistent temperature shifts that appear to be reproducible across host species. The effects of ambient temperature on gut microbiota in fish species have also been studied. In rainbow trout (*Oncorhynchus mykiss*), there is a negative relationship between rearing temperature and relative abundance of Firmicutes (Huyben et al., 2019). However, the relative abundance of Firmicutes and rearing temperature do not appear to be consistently connected or correlated in all fish species, which often have lower relative abundances of Firmicutes than larger relative abundances of Proteobacteria (Ley et al., 2008). The majority of the observed changes in the composition of fish gut microbiota in response to temperature fluctuations are mediated by alterations in the distribution and abundance of Proteobacterial linages. According to a recent study on the gut microbes of salmon (*Salmo salar*), rising temperatures were associated with shifts in the richness of Gamma proteobacterial linages, decreases in the abundances of *Acinetobacter*, and an increase in *Vibrio* species known to be pathogenic (Neuman et al., 2016). Similar changes in Gamma proteobacterial abundance were found in yellowtail kingfish (*Seriola lalandi*) too (Soriano et al., 2018).

## Future Research Work

Our knowledge of the microbiome’s impact on fish performance and health continues to advance at a rapid rate and future studies on teleost systems should focus on improving the favourable pro-endocrine microbiome for successful reproduction. The majority of existing research has been done on animals, and there is a significant need to understand how microbiomics might be used to regulate potential physiology, immunology, and reproductive health risks in fish. Additional new innovative studies must be focused on the following research areas:

• The role of microbes in male and female gametogenesis, reproductive organ maturation, development, sexual differentiation, and sex change mechanisms in teleosts.
• The neurobiological and behavioral effects of the microbiota in the fishes exposed to toxic chemicals/contaminants.
• How dysbiosis of the gut microbiota is linked to the onset and progression of neurological illnesses in fishes.
• To establish a comprehensive and mechanistic strategy to untangle microbiota-brain communication in order to develop microbiota-based therapeutics to treat any microbe-induced neurological disorder in fish.
• Develop a microbial consortium diet with the potential to treat metabolic disorders by targeting the microbiota-gut-brain axis (MGBA).
• How microbial metabolites interact with host neurotransmitters.

## Conclusion

Maintaining gut microbial balance is critical for fish health and reproductive fitness. Several fundamental studies have shown various potential ways by which bacteria may interact with host tissues to regulate their energy metabolism. The endocrine system and microbiota both influence physiological processes across systems in different ways. There is a significant amount of research demonstrating intricate, frequently bi-directional interactions between gut bacteria and host endocrine systems in other models. The majority of research on microbial interactions and the endocrine system is based exclusively on microbial sequencing technology, specifically. In these circumstances, metagenomics may be useful, but it has limitations due to its inability to assess *in situ* microbial activity. 16S rRNA amplicon sequencing, metagenomics, meta-transcriptomic, and whole genome metagenomics technique will make it easier to identify metabolic pathways that are responsible for the production of a wide range of microbial metabolites. The selection of probiotics to work properly, as well as the production of GABA and serotonin by probiotic bacteria, may reflect the largely underappreciated importance of neuroscience in understanding how microbes may influence health via both neuroimmune and neurophysiological mechanisms. In order to comprehend the complexities of microbiota-host interactions and the sophistication of disorders, the animal models studied require additional research to optimize targets and therapeutic approaches. This study will address a holistic approach towards new breeding technology and conservation endocrinology. This review will also provide an insight into microbiota-mediated manipulation of fish neurotransmission and its physiological implications and the mechanism that how neuroactive chemicals produced by gut microorganisms modify the gastrointestinal function and host behaviour via interconnecting the gut-brain interface. Finally, good communication between researchers and application biologists is required for this knowledge to be employed to maximize teleost health and reproduction.

## References

[B1] Abad M. E. Binkhorst F. M. P. Elbal M. T. Rombou J. H. W. M. (1987). A Comparative Immunocytochemical Study of the Gastro-Entero-Pancreatic (GEP) Endocrine System in a Stomachless and a Stomach-Containing Teleost. General Comp. Endocrinol. 66, 123–136. 10.1016/0016-6480(87)90357-1 2884163

[B2] Adlercreutz H. Pulkkinen M. O. Hämäläinen E. K. Korpela J. T. (1984). Studies on the Role of Intestinal Bacteria in Metabolism of Synthetic and Natural Steroid Hormones. J. Steroid Biochem. 20, 217–229. 10.1016/0022-4731(84)90208-5 6231418

[B3] Agrawal S. M. Omeljaniuk R. J. (2000). Levels of Specifically Bound [3H]ketanserin Compared with Levels of Serotonin (5HT) in the Brain Regions of Juvenile and Sexually Recrudescing Female Rainbow Trout, *Oncorhynchus mykiss* . Can. J. Physiol. Pharmacol. 78, 228–236. 10.1139/y99-135 10721814

[B4] Akash S. Shah P. Shafeeque M. Pooja A. S. Zacharia P. U. Ajith J. K. (2021). Observed Links between Coastal Ocean Processes and Indian Oil Sardine (Sardinella Longiceps) Fishery along the Southwest Coast of India. Regional Stud. Mar. Sci. 46, 101850. 10.1016/j.rsma.2021.101850

[B5] Andlid T. Juárez R.-V. Gustafsson L. (1995). Yeast Colonizing the Intestine of Rainbow Trout (Salmo Gairdneri) and Turbot (Scophtalmus Maximus). Microb. Ecol. 30, 321–334. 10.1007/BF00171938 24185568

[B6] Antwis R. E. Edwards K. L. Unwin B. Walker S. L. Shultz S. (2019). Rare Gut Microbiota Associated with Breeding Success, Hormone Metabolites and Ovarian Cycle Phase in the Critically Endangered Eastern Black Rhino. Microbiome 7, 27. 10.1186/s40168-019-0639-0 30770764 PMC6377766

[B7] Asano Y. Hiramoto T. Nishino R. Aiba Y. Kimura T. Yoshihara K. (2012). Critical Role of Gut Microbiota in the Production of Biologically Active, Free Catecholamines in the Gut Lumen of Mice. Am. J. Physiology-Gastrointestinal Liver Physiology 303, G1288–G1295. 10.1152/ajpgi.00341.2012 23064760

[B8] Avella M. A. Place A. Du S.-J. Williams E. Silvi S. Zohar Y. (2012). Lactobacillus Rhamnosus Accelerates Zebrafish Backbone Calcification and Gonadal Differentiation through Effects on the GnRH and IGF Systems. PLOS ONE 7, e45572. 10.1371/journal.pone.0045572 23029107 PMC3447769

[B9] Baj A. Moro E. Bistoletti M. Orlandi V. Crema F. Giaroni C. (2019). Glutamatergic Signaling along the Microbiota-Gut-Brain Axis. Int. J. Mol. Sci. 20, 1482. 10.3390/ijms20061482 PMC6471396 30934533

[B10] Bakke-McKellep A. M. Penn M. H. Salas P. M. Refstie S. Sperstad S. Landsverk T. (2007). Effects of Dietary Soyabean Meal, Inulin and Oxytetracycline on Intestinal Microbiota and Epithelial Cell Stress, Apoptosis and Proliferation in the Teleost Atlantic Salmon (*Salmo salar* L.). Br. J. Nutr. 97, 699–713. 10.1017/S0007114507381397 17349083

[B215] Balcazar J. L. Blas I. de. Zarzuela I. R. Cunningham D. Vendrell D. Muzquiz J. L. (2006). The Role of Probiotics in Aquaculture. Vet. Microbiol. 114, 173–186. 10.1016/j.vetmic.2006.01.009 16490324

[B11] Banerjee G. Ray A. K. (2017). Bacterial Symbiosis in the Fish Gut and its Role in Health and Metabolism. Symbiosis 72, 1–11. 10.1007/s13199-016-0441-8

[B12] Baquero F. Tedim A.-S. Coque T. (2013). Antibiotic Resistance Shaping Multi-Level Population Biology of Bacteria. Front. Microbiol. 4, 15. 10.3389/fmicb.2013.00015 23508522 PMC3589745

[B13] Barange M. Lauria V. Das I. Hazra S. Cazcarro I. Arto I. (2018). Importance of Fisheries for Food Security across Three Climate Change Vulnerable Deltas. Sci. Total Environ. 640–641, 1566–1577. 10.1016/j.scitotenv.2018.06.011 30021321

[B14] Barrett E. Ross R. p. O’Toole P. w. Fitzgerald G. f. Stanton C. (2012). γ-Aminobutyric Acid Production by Culturable Bacteria from the Human Intestine. J. Appl. Microbiol. 113, 411–417. 10.1111/j.1365-2672.2012.05344.x 22612585

[B15] Bastías R. Higuera G. Sierralta W. Espejo R. T. (2010). A New Group of Cosmopolitan Bacteriophages Induce a Carrier State in the Pandemic Strain of Vibrio Parahaemolyticus. Environ. Microbiol. 12, 990–1000. 10.1111/j.1462-2920.2010.02143.x 20105216

[B16] Baud D. Pattaroni C. Vulliemoz N. Castella V. Marsland B. J. Stojanov M. (2019). Sperm Microbiota and its Impact on Semen Parameters. Front. Microbiol. 10, 234. 10.3389/fmicb.2019.00234 30809218 PMC6379293

[B17] Baumgart D. C. Sandborn W. J. (2012). Crohn’s Disease. Lancet 380, 1590–1605. 10.1016/S0140-6736(12)60026-9 22914295

[B18] Bhari B. Visvanathan C. (2018). “Sustainable Aquaculture: Socio-Economic and Environmental Assessment,” in Sustainable Aquaculture (Cham: Springer), 63–93. 10.1007/978-3-319-73257-2_2

[B19] Bjurstöm H. Wang J. Ericsson I. Bengtsson M. Liu Y. Kumar-Mendu S. (2008). GABA, a Natural Immunomodulator of T Lymphocytes. J. Neuroimmunol. 205, 44–50. 10.1016/j.jneuroim.2008.08.017 18954912

[B20] Blache P. van de Wetering M. Duluc I. Domon C. Berta P. Freund J.-N. (2004). SOX9 Is an Intestine Crypt Transcription Factor, Is Regulated by the Wnt Pathway, and Represses the CDX2 and MUC2 Genes. J. Cell Biol. 166, 37–47. 10.1083/jcb.200311021 15240568 PMC2172132

[B21] Bolnick D. I. Snowberg L. K. Caporaso J. G. Lauber C. Knight R. Stutz W. E. (2014). Major Histocompatibility Complex Class IIb Polymorphism Influences Gut Microbiota Composition and Diversity. Mol. Ecol. 23, 4831–4845. 10.1111/mec.12846 24975397

[B22] Borrelli L. Aceto S. Agnisola C. De Paolo S. Dipineto L. Stilling R. M. (2016). Probiotic Modulation of the Microbiota-Gut-Brain axis and Behaviour in Zebrafish. Sci. Rep. 6, 30046. 10.1038/srep30046 27416816 PMC4945902

[B23] Boutin S. Sauvage C. Bernatchez L. Audet C. Derome N. (2014). Inter Individual Variations of the Fish Skin Microbiota: Host Genetics Basis of Mutualism? PLOS ONE 9, e102649. 10.1371/journal.pone.0102649 25068850 PMC4113282

[B24] Brugman S. (2016). The Zebrafish as a Model to Study Intestinal Inflammation. Dev. Comp. Immunol. 64, 82–92. 10.1016/j.dci.2016.02.020 26902932

[B25] Burns A. R. Stephens W. Z. Stagaman K. Wong S. Rawls J. F. Guillemin K. (2016). The Composition of the Zebrafish Intestinal Microbial Community Varies across Development. ISME J. 10, 644–654. 10.1038/ismej.2015.140 26339860 PMC4817687

[B26] Butt R. L. Volkoff H. (2019). Gut Microbiota and Energy Homeostasis in Fish. Front. Endocrinol. 10, 9. 10.3389/fendo.2019.00009 PMC6353785 30733706

[B27] Cahill D. G. Hamers R. J. (1990). Ultrafast Time Resolution in Scanned Probe Microscopies. Appl. Phys. Lett. 57, 2031–2033. 10.1063/1.103997

[B28] Cani P. D. Everard A. Duparc T. (2013). Gut Microbiota, Enteroendocrine Functions and Metabolism. Curr. Opin. Pharmacol. 13, 935–940. 10.1016/j.coph.2013.09.008 24075718

[B29] Caporaso J. G. Lauber C. L. Walters W. A. Berg-Lyons D. Huntley J. Fierer N. (2012). Ultra-high-throughput Microbial Community Analysis on the Illumina HiSeq and MiSeq Platforms. ISME J. 6, 1621–1624. 10.1038/ismej.2012.8 22402401 PMC3400413

[B30] Carnevali O. Avella M. A. Gioacchini G. (2013). Effects of Probiotic Administration on Zebrafish Development and Reproduction. General Comp. Endocrinol. 188, 297–302. 10.1016/j.ygcen.2013.02.022 23500006

[B31] Catalán N. Villasante A. Wacyk J. Ramírez C. Romero J. (2018). Fermented Soybean Meal Increases Lactic Acid Bacteria in Gut Microbiota of Atlantic Salmon (*Salmo salar*). Probiotics Antimicro. Prot. 10, 566–576. 10.1007/s12602-017-9366-7 29274013

[B32] Catania A. Colombo G. Rossi C. Carlin A. Sordi A. Lonati C. (2006). Antimicrobial Properties of α-MSH and Related Synthetic Melanocortins. TheScientificWorldJOURNAL 6, 1241–1246. 10.1100/tsw.2006.227 17028769 PMC5917254

[B33] Chi Z. Chi Z. Zhang T. Liu G. Yue L. (2009). Inulinase-expressing Microorganisms and Applications of Inulinases. Appl. Microbiol. Biotechnol. 82, 211–220. 10.1007/s00253-008-1827-1 19122997

[B34] Chu H. Shen C. Xiong J. Zhang H. Feng Y. Lin X. (2013). Soil pH Drives the Spatial Distribution of Bacterial Communities along Elevation on Changbai Mountain. Soil Biol. Biochem. 57, 204–211. 10.1016/j.soilbio.2012.07.013

[B35] Cicala F. Lago-Leston A. Gomez-Gil B. Gollas T. Chong J. Cortes-Jacinto E. (2020). Gut Microbiota Shifts in the Giant Tiger Shrimp, *Penaeus monodon*, during the Postlarvae, Juvenile, and Adult Stages. Aquac. Int. 28, 1421–1433. 10.1007/s10499-020-00532-1

[B36] Clarke G. Grenham S. Scully P. Fitzgerald P. Moloney R. D. Shanahan F. (2013). The Microbiome-Gut-Brain axis during Early Life Regulates the Hippocampal Serotonergic System in a Sex-dependent Manner. Mol. Psychiatry 18, 666–673. 10.1038/mp.2012.77 22688187

[B37] Clarke G. Stilling R. M. Kennedy P. J. Stanton C. Cryan J. F. Dinan T. G. (2014). Minireview: Gut Microbiota: The Neglected Endocrine Organ. Mol. Endocrinol. 28, 1221–1238. 10.1210/me.2014-1108 24892638 PMC5414803

[B38] Clelland E. Peng C. Tan Q. (2009). Potential Role of Bone Morphogenetic Protein-15 in Zebrafish Follicle Development and Oocyte Maturation. Comp. Biochem. Physiology Part A Mol. Integr. Physiology 153, 83–87. 10.1016/j.cbpa.2008.09.034 18951993

[B39] Clemente J. C. Ursell L. K. Parfrey L. W. Knight R. (2012). The Impact of the Gut Microbiota on Human Health: An Integrative View. Cell 148, 1258–1270. 10.1016/j.cell.2012.01.035 22424233 PMC5050011

[B40] Clements K. D. Angert E. R. Montgomery W. L. Choat J. H. (2014). Intestinal Microbiota in Fishes: What’s Known and What’s Not. Mol. Ecol. 23, 1891–1898. 10.1111/mec.12699 24612310

[B41] Clements K. D. Pasch I. B. Y. Moran D. Turner S. J. (2007). Clostridia Dominate 16S rRNA Gene Libraries Prepared from the Hindgut of Temperate Marine Herbivorous Fishes. Mar. Biol. 150, 1431–1440. 10.1007/s00227-006-0443-9

[B42] Collins S. M. Bakan C. E. Swartzel G. D. Hofmeister C. C. Efebera Y. A. Kwon H. (2013). Elotuzumab Directly Enhances NK Cell Cytotoxicity against Myeloma via CS1 Ligation: Evidence for Augmented NK Cell Function Complementing ADCC. Cancer Immunol. Immunother. 62, 1841–1849. 10.1007/s00262-013-1493-8 24162108 PMC4134870

[B43] Cryan J. F. Sherwin E. Dinan T. G. (2018). Recent Developments in Understanding the Role of the Gut Microbiota in Brain Health and Disease. Ann. N. Y. Acad. Sci. 1420, 5–25. 10.1111/nyas.13416 28768369

[B213] Curson A. R. J. Sullivan M. J. Todd J. D. AJohntson A. W. B. (2010). Identification of Genes for Dimethyl Sulfide Production in Bacteria in the Gut of Atlantic Herring (*Clupea harengus*). International Society for Microbial Ecology 4, 144–146. 10.1038/ismej.2009.93 19710707

[B44] Das P. Mandal S. Khan A. Manna S. K. Ghosh K. (2014). Distribution of Extracellular Enzyme-Producing Bacteria in the Digestive Tracts of 4 Brackish Water Fish Species. Turk J. Zool. 38, 79–88.

[B45] Davis D. J. Bryda E. C. Gillespie C. H. Ericsson A. C. (2016). Microbial Modulation of Behavior and Stress Responses in Zebrafish Larvae. Behav. Brain Res. 311, 219–227. 10.1016/j.bbr.2016.05.040 27217102 PMC6423445

[B46] Dawood M. A. O. (2021). Nutritional Immunity of Fish Intestines: Important Insights for Sustainable Aquaculture. Rev. Aquac. 13, 642–663. 10.1111/raq.12492

[B47] Dehler C. E. Secombes C. J. Martin S. A. M. (2017). Environmental and Physiological Factors Shape the Gut Microbiota of Atlantic Salmon Parr (*Salmo salar* L.). Aquaculture 467, 149–157. 10.1016/j.aquaculture.2016.07.017 28111483 PMC5142738

[B48] Dinan T. G. Cussotto S. Sandhu K. V. Cryan J. F. (2018). The Neuroendocrinology of the Microbiota-Gut-Brain Axis: A Behavioural Perspective. Front. Neuroendocrinol. 51, 80–101. 10.1016/j.yfrne.2018.04.002 29753796

[B49] Dionne M. Miller K. M. Dodson J. J. Caron F. Bernatchez L. (2007). Clinal Variation in Mhc Diversity with Temperature: Evidence for the Role of Host–Pathogen Interaction on Local Adaptation in Atlantic Salmon. Evolution 61, 2154–2164. 10.1111/j.1558-5646.2007.00178.x 17767587

[B50] Donadio V. Nolano M. Provitera V. Perretti A. Stancanelli A. Saltalamacchia A. M. (2006). Ross Syndrome: a Rare or a Misknown Disorder of Thermoregulation? A Skin Innervation Study on 12 Subjects. Brain 129, 2119–2131. 10.1093/brain/awl175 16837483

[B51] Durant J. M. Hjermann D. Ø. Ottersen G. Stenseth N. C. (2007). Climate and the Match or Mismatch between Predator Requirements and Resource Availability. Clim. Res. 33, 271–283. 10.3354/cr033271

[B52] El Karim I. A. Linden G. J. Orr D. F. Lundy F. T. (2008). Antimicrobial Activity of Neuropeptides against a Range of Micro-organisms from Skin, Oral, Respiratory and Gastrointestinal Tract Sites. J. Neuroimmunol. 200, 11–16. 10.1016/j.jneuroim.2008.05.014 18603306

[B53] Elahi S. Ertelt J. M. Kinder J. M. Jiang T. T. Zhang X. Xin L. (2013). Immunosuppressive CD71+ Erythroid Cells Compromise Neonatal Host Defence against Infection. Nature 504, 158–162. 10.1038/nature12675 24196717 PMC3979598

[B54] Erny D. Kierdorf K. Goldmann T. Sander V. Schulz C. Perdiguero E. G. (2013). Microglia Emerge from Erythromyeloid Precursors via Pu.1- and Irf8-dependent Pathways. Nat. Neurosci. 16, 273–280. 10.1038/nn.3318 23334579

[B55] Evans J. Morris L. Marchesi J. (2013). The Gut Microbiome: The Role of a Virtual Organ in the Endocrinology of the Host. J. Endocrinol. 218. 10.1530/JOE-13-0131 23833275

[B56] Evans M. L. Neff B. D. (2009). Major Histocompatibility Complex Heterozygote Advantage and Widespread Bacterial Infections in Populations of Chinook Salmon (*Oncorhynchus tshawytscha*). Mol. Ecol. 18, 4716–4729. 10.1111/j.1365-294X.2009.04374.x 19821902

[B212] FAO (2018). The State of World Fisheries and Aquaculture. Rome: Meeting the Sustainable Development Goals. CCBY-NC-SA 3.0 IGO

[B211] FAO (2020). The State of World Fisheries and Aquaculture 2020. Rome: Sustainability in Action. 10.4060/ca9229en

[B57] Fidopiastis P. M. Bezdek D. J. Horn M. H. Kandel J. S. (2006). Characterizing the Resident, Fermentative Microbial Consortium in the Hindgut of the Temperate-Zone Herbivorous Fish, Hermosilla Azurea (Teleostei: Kyphosidae). Mar. Biol. 148, 631–642. 10.1007/s00227-005-0106-2

[B58] Fierer N. Jackson R. B. (2006). The Diversity and Biogeography of Soil Bacterial Communities. PNAS 103, 626–631. 10.1073/pnas.0507535103 16407148 PMC1334650

[B59] Forsythe P. Kunze W. A. (2013). Voices from within: Gut Microbes and the CNS. Cell. Mol. Life Sci. 70, 55–69. 10.1007/s00018-012-1028-z 22638926 PMC11113561

[B60] Forsythe P. Sudo N. Dinan T. Taylor V. H. Bienenstock J. (2010). Mood and Gut Feelings. Brain, Behav. Immun. 24, 9–16. 10.1016/j.bbi.2009.05.058 19481599

[B61] Franchimont D. (2004). Overview of the Actions of Glucocorticoids on the Immune Response: A Good Model to Characterize New Pathways of Immunosuppression for New Treatment Strategies. Ann. N. Y. Acad. Sci. 1024, 124–137. 10.1196/annals.1321.009 15265777

[B62] Frank J. G. Kamendi H. W. Cheng Q. Dergacheva O. Gorini C. Jameson H. S. (2008). Recruitment of Excitatory Serotonergic Neurotransmission to Cardiac Vagal Neurons in the Nucleus Ambiguus Post Hypoxia and Hypercapnia. J. Neurophysiology 99, 1163–1168. 10.1152/jn.01178.2007 18184887

[B63] Furness J. B. (2016). “Integrated Neural and Endocrine Control of Gastrointestinal Function,” in The Enteric Nervous System: 30 Years Later Advances in Experimental Medicine and Biology. Editors Brierley S. Costa M. (Cham: Springer International Publishing), 159–173. 10.1007/978-3-319-27592-5_16 27379644

[B64] Ganapathy V. Shekhawat P. S. Sonne S. Carter A. L. Matern D. (2013). Enzymes Involved in L-Carnitine Biosynthesis Are Expressed by Small Intestinal Enterocytes in Mice: Implications for Gut Health. J. Crohn’s Colitis 7, e197–e205. 10.1016/j.crohns.2012.08.011 22999781 PMC3644392

[B65] Garcia-Reyero N. Williams C. L. Martyniuk C. J. Tubbs C. W. Bisesi J. H. (2020). Regulation of Endocrine Systems by the Microbiome: Perspectives from Comparative Animal Models. General Comp. Endocrinol. 292, 113437. 10.1016/j.ygcen.2020.113437 32061639

[B66] Gatesoupe F. J. (2007). Live Yeasts in the Gut: Natural Occurrence, Dietary Introduction, and Their Effects on Fish Health and Development. Aquaculture 267, 20–30. 10.1016/j.aquaculture.2007.01.005

[B67] Gause W. C. Maizels R. M. (2016). Macrobiota — Helminths as Active Participants and Partners of the Microbiota in Host Intestinal Homeostasis. Curr. Opin. Microbiol. 32, 14–18. 10.1016/j.mib.2016.04.004 27116368 PMC4983462

[B68] Genton L. Kudsk K. A. (2003). Interactions between the Enteric Nervous System and the Immune System: Role of Neuropeptides and Nutrition. Am. J. Surg. 186, 253–258. 10.1016/S0002-9610(03)00210-1 12946828

[B69] Ghanbari M. Kneifel W. Domig K. J. (2015). A New View of the Fish Gut Microbiome: Advances from Next-Generation Sequencing. Aquaculture 448, 464–475. 10.1016/j.aquaculture.2015.06.033

[B70] Giacomin P. Zakrzewski M. Croese J. Su X. Sotillo J. McCann L. (2015). Experimental Hookworm Infection and Escalating Gluten Challenges Are Associated with Increased Microbial Richness in Celiac Subjects. Sci. Rep. 5, 13797. 10.1038/srep13797 26381211 PMC4585380

[B71] Gioacchini G. (2011). Effects of Probiotic on Zebrafish Reproduction. J. Aquac. Res. Dev. s1. 10.4172/2155-9546.S1-002

[B72] Gioacchini G. Giorgini E. Olivotto I. Maradonna F. Merrifield D. L. Carnevali O. (2014). The Influence of Probiotics on Zebrafish Danio Rerio Innate Immunity and Hepatic Stress. Zebrafish 11, 98–106. 10.1089/zeb.2013.0932 24564619

[B73] Givens C. E. Ransom B. Bano N. Hollibaugh J. T. (2015). Comparison of the Gut Microbiomes of 12 Bony Fish and 3 Shark Species. Mar. Ecol. Prog. Ser. 518, 209–223. 10.3354/meps11034

[B74] Gómez G. D. Balcázar J. L. (2008). A Review on the Interactions between Gut Microbiota and Innate Immunity of Fish. FEMS Immunol. Med. Microbiol. 52, 145–154. 10.1111/j.1574-695X.2007.00343.x 18081845

[B75] Grahame-Smith D. G. (1967). The Biosynthesis of 5-hydroxytryptamine in Brain. Biochem. J. 105, 351–360. 10.1042/bj1050351 6056632 PMC1198307

[B76] Grice E. A. Segre J. A. (2012). The Human Microbiome: Our Second Genome. Annu. Rev. Genomics Hum. Genet. 13, 151–170. 10.1146/annurev-genom-090711-163814 22703178 PMC3518434

[B77] Gupta S. Fernandes J. Kiron V. (2019). Antibiotic-Induced Perturbations Are Manifested in the Dominant Intestinal Bacterial Phyla of Atlantic Salmon. Microorganisms 7, 233. 10.3390/microorganisms7080233 PMC6723382 31382431

[B78] Hammer T. J. Moran N. A. Ochman H. (2019). Evolutionary and Ecological Consequences of Gut Microbial Communities. Annu. Rev. Ecol. Evol. Syst. 50, 451–475. 10.1146/annurev-ecolsys-110617-062453 32733173 PMC7392196

[B79] Haygood A. M. Jha R. (2018). Strategies to Modulate the Intestinal Microbiota of Tilapia (Oreochromis sp.) in Aquaculture: a Review. Rev. Aquac. 10, 320–333. 10.1111/raq.12162

[B80] Heiman M. L. Greenway F. L. (2016). A Healthy Gastrointestinal Microbiome Is Dependent on Dietary Diversity. Mol. Metab. 5, 317–320. 10.1016/j.molmet.2016.02.005 27110483 PMC4837298

[B81] Hernandez L. P. Farina S. C. Kane E. A. (2019). Multifunctional Structures and Multistructural Functions: Integration in the Evolution of Biomechanical Systems. Integr. Comp. Biol. 59, 338–345. 10.1093/icb/icz095 31168594

[B82] Holmgren S. Jönsson A.-C. Holstein B. (1986). “Gastro-Intestinal Peptides in Fish,” in Fish Physiology: Recent Advances. Editors Nilsson S. Holmgren S. (Dordrecht: Springer Netherlands), 119–139. 10.1007/978-94-011-6558-7_7

[B83] Holst J. J. Fahrenkrug J. Stadil F. Rehfeld J. F. (1996). Gastrointestinal Endocrinology. Scand. J. Gastroenterology 31, 27–38. 10.3109/00365529609094558 8726276

[B84] Holub B. S. Rauch I. Radner S. Sperl W. Hell M. Kofler B. (2011). Effects of Galanin Message-Associated Peptide and Neuropeptide Y against Various Non-albicans Candida Strains. Int. J. Antimicrob. Agents 38, 76–80. 10.1016/j.ijantimicag.2011.02.019 21550784

[B85] Holzer P. Reichmann F. Farzi A. (2012). Neuropeptide Y, Peptide YY and Pancreatic Polypeptide in the Gut–Brain axis. Neuropeptides 46, 261–274. 10.1016/j.npep.2012.08.005 22979996 PMC3516703

[B86] Hoseinifar S. H. Ringø E. Shenavar Masouleh A. Esteban M. Á. (2016). Probiotic, Prebiotic and Synbiotic Supplements in Sturgeon Aquaculture: a Review. Rev. Aquac. 8, 89–102. 10.1111/raq.12082

[B87] Hovda M. B. Fontanillas R. McGurk C. Obach A. Rosnes J. T. (2012). Seasonal Variations in the Intestinal Microbiota of Farmed Atlantic Salmon (*Salmo salar* L.): Seasonal Variations in the Intestinal Microbiota of *Salmo salar* L. Aquac. Res. 43, 154–159. 10.1111/j.1365-2109.2011.02805.x

[B88] Hughes D. T. Sperandio V. (2008). Inter-kingdom Signalling: Communication between Bacteria and Their Hosts. Nat. Rev. Microbiol. 6, 111–120. 10.1038/nrmicro1836 18197168 PMC2667375

[B89] Huyben D. Vidaković A. Werner Hallgren S. Langeland M. (2019). High-throughput Sequencing of Gut Microbiota in Rainbow Trout (*Oncorhynchus mykiss*) Fed Larval and Pre-pupae Stages of Black Soldier Fly (Hermetia Illucens). Aquaculture 500, 485–491. 10.1016/j.aquaculture.2018.10.034

[B90] Infante-Villamil S. Huerlimann R. Jerry D. R. (2021). Microbiome Diversity and Dysbiosis in Aquaculture. Rev. Aquac. 13, 1077–1096. 10.1111/raq.12513

[B91] Ito K. Tanaka K. Nishibe Y. Hasegawa J. Ueno H. (2007). GABA-Synthesizing Enzyme, GAD67, from Dermal Fibroblasts: Evidence for a New Skin Function. Biochimica Biophysica Acta (BBA) - General Subj. 1770, 291–296. 10.1016/j.bbagen.2006.09.017 17113713

[B92] Itoi S. Okamura T. Koyama Y. Sugita H. (2006). Chitinolytic Bacteria in the Intestinal Tract of Japanese Coastal Fishes. Can. J. Microbiol. 52, 1158–1163. 10.1139/w06-082 17473885

[B93] Iyer L. M. Balaji S. Babu M. M. Aravind L. (2005). Discovery of the Principal Specific Transcription Factors of Apicomplexa and Their Implication for the Evolution of the AP2-Integrase DNA Binding Domains. Nucleic Acids Res. 33, 3994–4006. 10.1093/nar/gki709 16040597 PMC1178005

[B94] Kanther M. Rawls J. F. (2010). Host–microbe Interactions in the Developing Zebrafish. Curr. Opin. Immunol. 22, 10–19. 10.1016/j.coi.2010.01.006 20153622 PMC3030977

[B95] Kapatral V. Ivanova N. Anderson I. Reznik G. Bhattacharyya A. Gardner W. L. (2003). Genome Analysis of F. Nucleatum Sub Spp Vincentii and its Comparison with the Genome of F. Nucleatum ATCC 25586. Genome Res. 13, 1180–1189. 10.1101/gr.566003 12799352 PMC403646

[B96] Karavolos M. H. Winzer K. Williams P. Khan C. M. A. (2013). Pathogen Espionage: Multiple Bacterial Adrenergic Sensors Eavesdrop on Host Communication Systems. Mol. Microbiol. 87, 455–465. 10.1111/mmi.12110 23231070

[B97] Khan I. A. Thomas P. (1992). Stimulatory Effects of Serotonin on Maturational Gonadotropin Release in the Atlantic Croaker, *Micropogonias undulatus* . General Comp. Endocrinol. 88, 388–396. 10.1016/0016-6480(92)90233-A 1490584

[B98] Kim D.-H. Brunt J. Austin B. (2007). Microbial Diversity of Intestinal Contents and Mucus in Rainbow Trout (*Oncorhynchus mykiss*). J. Appl. Microbiol. 102, 1654–1664. 10.1111/j.1365-2672.2006.03185.x 17578431

[B99] Kohli G. Clelland E. Peng C. (2005). Potential Targets of Transforming Growth Factor-Beta1 during Inhibition of Oocyte Maturation in Zebrafish. Reprod. Biol. Endocrinol. 3, 53. 10.1186/1477-7827-3-53 16197550 PMC1274345

[B100] Kornman K. S. Loesche W. J. (1982). Effects of Estradiol and Progesterone on Bacteroides Melaninogenicus and Bacteroides Gingivalis. Infect. Immun. 35, 256–263. 10.1128/iai.35.1.256-263.1982 6119293 PMC351023

[B101] Kovatcheva-Datchary P. Arora T. (2013). Nutrition, the Gut Microbiome and the Metabolic Syndrome. Best Pract. Res. Clin. Gastroenterology 27, 59–72. 10.1016/j.bpg.2013.03.017 23768553

[B102] Kumar R. Mukherjee S. C. Prasad K. P. Pal A. K. (2006). Evaluation of Bacillus Subtilis as a Probiotic to Indian Major Carp Labeo Rohita (Ham.). Aquac. Res. 37, 1215–1221. 10.1111/j.1365-2109.2006.01551.x

[B103] Kumar R. Mukherjee S. C. Ranjan R. Nayak S. K. (2008). Enhanced Innate Immune Parameters in Labeo Rohita (Ham.) Following Oral Administration of Bacillus Subtilis. Fish Shellfish Immunol. 24, 168–172. 10.1016/j.fsi.2007.10.008 18060807

[B104] Kumar R. Mukherjee S. C. Ranjan R. Vani T. Brahmachari R. K. Nayak S. K. (2015). Effect of Dietary Supplementation of Bacillus Subtilis on Haematological and Immunological Parameters of Catla Catla (Hamilton). Aquacult Int. 23, 1275–1292. 10.1007/s10499-015-9883-x

[B105] Kunc M. Gabrych A. Witkowski J. M. (2016). Microbiome Impact on Metabolism and Function of Sex, Thyroid, Growth and Parathyroid Hormones. Acta Biochim. Pol. 63, 189–201. 10.18388/abp.2015_1093 26505128

[B106] Kwa M. Plottel C. S. Blaser M. J. Adams S. (2016). The Intestinal Microbiome and Estrogen Receptor–Positive Female Breast Cancer. JNCI J. Natl. Cancer Inst. 108, djw029. 10.1093/jnci/djw029 PMC5017946 27107051

[B107] Lam S. H. Chua H. L. Gong Z. Lam T. J. Sin Y. M. (2004). Development and Maturation of the Immune System in Zebrafish, *Danio rerio*: a Gene Expression Profiling, *In Situ* Hybridization and Immunological Study. Dev. Comp. Immunol. 28, 9–28. 10.1016/S0145-305X(03)00103-4 12962979

[B108] Leeuw R. D. Habibi H. R. Nahorniak C. S. Peter R. E. (1989). Dopaminergic Regulation of Pituitary Gonadotrophin-Releasing Hormone Receptor Activity in the Goldfish (*Carassius auratus*). J. Endocrinol. 121, 239–247. 10.1677/joe.0.1210239 2547004

[B109] Legrand T. P. R. A. Wynne J. W. Weyrich L. S. Oxley A. P. A. (2020). A Microbial Sea of Possibilities: Current Knowledge and Prospects for an Improved Understanding of the Fish Microbiome. Rev. Aquac. 12, 1101–1134. 10.1111/raq.12375

[B110] Ley R. E. Godoy-Vitorino F. Gao Z. Pei Z. Ortiz-Zuazaga H. Pericchi L. R. (2008). Bacterial Community in the Crop of the Hoatzin, a Neotropical Folivorous Flying Bird. Appl. Environ. Microbiol. 74, 5905–5912. 10.1128/AEM.00574-08 18689523 PMC2565963

[B111] Li M. Penner G. b. Hernandez-Sanabria E. Oba M. Guan L. l. (2009). Effects of Sampling Location and Time, and Host Animal on Assessment of Bacterial Diversity and Fermentation Parameters in the Bovine Rumen. J. Appl. Microbiol. 107, 1924–1934. 10.1111/j.1365-2672.2009.04376.x 19508296

[B112] Liu H.-H. Zhou S.-S. Xu J. Zhu H. Wu J. Xu J.-D. (2016). Gut Microbiota-Involved Mechanisms in Enhancing Systemic Exposure of Ginsenosides by Coexisting Polysaccharides in Ginseng Decoction. Sci. Rep. 6, 22474. 10.1038/srep22474 26932472 PMC4774164

[B113] Llewellyn M. S. McGinnity P. Dionne M. Letourneau J. Thonier F. Carvalho G. R. (2016). The Biogeography of the Atlantic Salmon (*Salmo salar*) Gut Microbiome. ISME J. 10, 1280–1284. 10.1038/ismej.2015.189 26517698 PMC5029221

[B114] Longo G. Bernardi G. (2015). The Evolutionary History of the Embiotocid Surfperch Radiation Based on Genome-wide RAD Sequence Data. Mol. Phylogenetics Evol. 88, 55–63. 10.1016/j.ympev.2015.03.027 25858559

[B115] Louis P. Hold G. L. Flint H. J. (2014). The Gut Microbiota, Bacterial Metabolites and Colorectal Cancer. Nat. Rev. Microbiol. 12, 661–672. 10.1038/nrmicro3344 25198138

[B116] Lozupone C. A. Knight R. (2007). Global Patterns in Bacterial Diversity. PNAS 104, 11436–11440. 10.1073/pnas.0611525104 17592124 PMC2040916

[B117] Lustri B. C. Sperandio V. Moreira C. G. (2017). Bacterial Chat: Intestinal Metabolites and Signals in Host-Microbiota-Pathogen Interactions. Infect. Immun. 85, e00476–17. 10.1128/IAI.00476-17 28947641 PMC5695128

[B118] Lyte J. M. Proctor A. Phillips G. J. Lyte M. Wannemuehler M. (2019). Altered Schaedler Flora Mice: A Defined Microbiota Animal Model to Study the Microbiota-Gut-Brain axis. Behav. Brain Res. 356, 221–226. 10.1016/j.bbr.2018.08.022 30153465

[B119] Lyte M. Ernst S. (1992). Catecholamine Induced Growth of Gram Negative Bacteria. Life Sci. 50, 203–212. 10.1016/0024-3205(92)90273-R 1731173

[B120] Lyte M. (2014). Microbial Endocrinology. Gut Microbes 5, 381–389. 10.4161/gmic.28682 24690573 PMC4153777

[B121] Lyte M. (2013). Microbial Endocrinology in the Microbiome-Gut-Brain Axis: How Bacterial Production and Utilization of Neurochemicals Influence Behavior. PLOS Pathog. 9, e1003726. 10.1371/journal.ppat.1003726 24244158 PMC3828163

[B122] Lyte M. (2010a). “Microbial Endocrinology: A Personal Journey,” in Microbial Endocrinology: Interkingdom Signaling in Infectious Disease and Health. Editors Lyte M. Freestone P. P. E. (New York, NY: Springer), 1–16. 10.1007/978-1-4419-5576-0_1

[B123] Lyte M. (2010b). The Microbial Organ in the Gut as a Driver of Homeostasis and Disease. Med. Hypotheses 74, 634–638. 10.1016/j.mehy.2009.10.025 19900764

[B124] Lyte M. (1993). The Role of Microbial Endocrinology in Infectious Disease. J. Endocrinol. 137, 343–345. 10.1677/joe.0.1370343 8371072

[B125] MacDonald N. L. Stark J. R. Austin B. (1986). Bacterial Microflora in the Gastro-Intestinal Tract of Dover Sole (*Solea solea* L.), with Emphasis on the Possible Role of Bacteria in the Nutrition of the Host. FEMS Microbiol. Lett. 35, 107–111.

[B126] Maji A. Misra R. Dhakan D. B. Gupta V. Mahato N. K. Saxena R. (2018). Gut Microbiome Contributes to Impairment of Immunity in Pulmonary Tuberculosis Patients by Alteration of Butyrate and Propionate Producers. Environ. Microbiol. 20, 402–419. 10.1111/1462-2920.14015 29322681

[B127] Marchant T. A. Peter R. E. (1986). Seasonal Variations in Body Growth Rates and Circulating Levels of Growth Hormone in the Goldfish, *Carassius auratus* . J. Exp. Zoology 237, 231–239. 10.1002/jez.1402370209 3950566

[B128] Martin E. Weger B. D. Gobet C. Yeung J. Jimenez S. Betrisey B. (2019). The Mouse Microbiome Is Required for Sex-specific Diurnal Rhythms of Gene Expression and Metabolism. Cell Metab. 29, 362–382. e8. 10.1016/j.cmet.2018.09.023 30344015 PMC6370974

[B129] McFall-Ngai M. Hadfield M. G. Bosch T. C. G. Carey H. V. Domazet-Lošo T. Douglas A. E. (2013). Animals in a Bacterial World, a New Imperative for the Life Sciences. PNAS 110, 3229–3236. 10.1073/pnas.1218525110 23391737 PMC3587249

[B130] Menon K. Bajbouj K. Shafarin J. Muhammad J. S. Ali A. Unnikannan H. (2020). Estrogen Signaling Differentially Alters Iron Metabolism in Monocytes in an Interleukin 6-dependent Manner. Immunobiology 225, 151995. 10.1016/j.imbio.2020.151995 32962815

[B131] Merrifield D. L. Rodiles A. (2015). “10 - the Fish Microbiome and its Interactions with Mucosal Tissues,” in Mucosal Health in Aquaculture. Editors Beck B. H. Peatman E. (San Diego: Academic Press), 273–295. 10.1016/B978-0-12-417186-2.00010-8

[B132] Messaoudi M. Lalonde R. Violle N. Javelot H. Desor D. Nejdi A. (2011). Assessment of Psychotropic-like Properties of a Probiotic Formulation (Lactobacillus Helveticus R0052 and Bifidobacterium Longum R0175) in Rats and Human Subjects. Br. J. Nutr. 105, 755–764. 10.1017/S0007114510004319 20974015

[B133] Méthot P.-O. Alizon S. (2014). What Is a Pathogen? toward a Process View of Host-Parasite Interactions. Virulence 5, 775–785. 10.4161/21505594.2014.960726 25483864 PMC4601502

[B134] Miller K. E. Hoffman E. M. Sutharshan M. Schechter R. (2011). Glutamate Pharmacology and Metabolism in Peripheral Primary Afferents: Physiological and Pathophysiological Mechanisms. Pharmacol. Ther. 130, 283–309. 10.1016/j.pharmthera.2011.01.005 21276816 PMC5937940

[B135] Miller K. N. Burhans M. S. Clark J. P. Howell P. R. Polewski M. A. DeMuth T. M. (2017). Aging and Caloric Restriction Impact Adipose Tissue, Adiponectin, and Circulating Lipids. Aging Cell 16, 497–507. 10.1111/acel.12575 28156058 PMC5418198

[B136] Miura T. Yamauchi K. Takahashi H. Nagahama Y. (1992). The Role of Hormones in the Acquisition of Sperm Motility in Salmonid Fish. J. Exp. Zoology 261, 359–363. 10.1002/jez.1402610316 1321204

[B137] Mohanta L. Das B. C. Patri M. (2020). Microbial Communities Modulating Brain Functioning and Behaviors in Zebrafish: A Mechanistic Approach. Microb. Pathog. 145, 104251. 10.1016/j.micpath.2020.104251 32418919

[B138] Moloney R. D. Desbonnet L. Clarke G. Dinan T. G. Cryan J. F. (2014). The Microbiome: Stress, Health and Disease. Mamm. Genome 25, 49–74. 10.1007/s00335-013-9488-5 24281320

[B139] Montalban-Arques A. De Schryver P. Bossier P. Gorkiewicz G. Mulero V. Gatlin D. M. (2015). Selective Manipulation of the Gut Microbiota Improves Immune Status in Vertebrates. Front. Immunol. 6, 512. 10.3389/fimmu.2015.00512 26500650 PMC4598590

[B140] Moreno I. Simon C. (2019). Deciphering the Effect of Reproductive Tract Microbiota on Human Reproduction. Reproductive Med. Biol. 18, 40–50. 10.1002/rmb2.12249 PMC6332752 30655720

[B141] Moschos S. Chan J. L. Mantzoros C. S. (2002). Leptin and Reproduction: a Review. Fertil. Steril. 77, 433–444. 10.1016/S0015-0282(01)03010-2 11872190

[B142] Nakano K. Ackerman P. Iwama G. K. Afonso L. O. B. Todgham A. (2004). Are Hsps Suitable for Indicating Stressed States in Fish? J. Exp. Biol. 207, 15–19. 10.1242/jeb.00707 14638828

[B143] Navarrete P. Espejo R. T. Romero J. (2008). Molecular Analysis of Microbiota along the Digestive Tract of Juvenile Atlantic Salmon (*Salmo salar* L.). Microb. Ecol. 57, 550. 10.1007/s00248-008-9448-x 18797955

[B144] Nayak S. K. (2010). Role of Gastrointestinal Microbiota in Fish. Aquac. Res. 41, 1553–1573. 10.1111/j.1365-2109.2010.02546.x

[B145] Neuman C. Hatje E. Zarkasi K. Z. Smullen R. Bowman J. P. Katouli M. (2016). The Effect of Diet and Environmental Temperature on the Faecal Microbiota of Farmed Tasmanian Atlantic Salmon (*Salmo salar* L.). Aquac. Res. 47, 660–672. 10.1111/are.12522

[B146] Nigam R. El-Nour H. Amatya B. Nordlind K. (2010). GABA and GABAA Receptor Expression on Immune Cells in Psoriasis: a Pathophysiological Role. Arch. Dermatol Res. 302, 507–515. 10.1007/s00403-010-1052-5 20455067

[B147] Obata K. Fogarty M. J. Smallcombe K. L. Yanagawa Y. Bellingham M. C. Noakes P. G. (2013). Genetic Deficiency of GABA Differentially Regulates Respiratory and Non-respiratory Motor Neuron Development. PLOS ONE 8, e56257. 10.1371/journal.pone.0056257 23457538 PMC3574162

[B148] Omar S. S. (2012). The Evaluation of Novel Bio-Ethanol Derived Co-products as Potential Feed Ingredients for Carp *Cyprinus carpio* and tilapia *Oreochromis niloticus* . Available at: https://pearl.plymouth.ac.uk/handle/10026.1/902 (Accessed December 6, 2021).

[B149] Omeljaniuk R. J. Shih S. H. Peter R. E. (1987). *In-vivo* Evaluation of Dopamine Receptor-Mediated Inhibition of Gonadotrophin Secretion from the Pituitary Gland of the Goldfish, *Carassius auratus* . J. Endocrinol. 114, 449–458. 10.1677/joe.0.1140449 2889788

[B150] Onarheim A. M. Wiik R. Burghardt J. Stackebrandt E. (1994). Characterization and Identification of Two Vibrio Species Indigenous to the Intestine of Fish in Cold Sea Water; Description of Vibrio Iliopiscarius Sp. Nov. Syst. Appl. Microbiol. 17, 370–379. 10.1016/S0723-2020(11)80053-6

[B151] Pankhurst N. W. (2011). The Endocrinology of Stress in Fish: An Environmental Perspective. General Comp. Endocrinol. 170, 265–275. 10.1016/j.ygcen.2010.07.017 20688064

[B152] Pavey S. A. Sevellec M. Adam W. Normandeau E. Lamaze F. C. Gagnaire P.-A. (2013). Nonparallelism in MHCIIβ Diversity Accompanies Nonparallelism in Pathogen Infection of Lake Whitefish (*Coregonus clupeaformis*) Species Pairs as Revealed by Next-Generation Sequencing. Mol. Ecol. 22, 3833–3849. 10.1111/mec.12358 23786238

[B153] Persicke M. Albersmeier A. Bednarz H. Niehaus K. Kalinowski J. Rückert C. (2015). Genome Sequence of the Soil Bacterium Corynebacterium Callunae Type Strain DSM 20147T. Stand. Genomic Sci. 10, 5. 10.1186/1944-3277-10-5 26203323 PMC4510995

[B154] Peter R. E. Lin H.-R. Van Der Kraak G. (1988). Induced Ovulation and Spawning of Cultured Freshwater Fish in China: Advances in Application of GnRH Analogues and Dopamine Antagonists. Aquaculture 74, 1–10. 10.1016/0044-8486(88)90080-4

[B155] Pond M. J. Stone D. M. Alderman D. J. (2006). Comparison of Conventional and Molecular Techniques to Investigate the Intestinal Microflora of Rainbow Trout (*Oncorhynchus mykiss*). Aquaculture 261, 194–203. 10.1016/j.aquaculture.2006.06.037

[B156] Quirk P. L. Siegel R. E. (2005). The Serotonin Type 3A Receptor Facilitates Luteinizing Hormone Release and LHβ Promoter Activity in Immortalized Pituitary Gonadotropes. Endocr 27, 37–43. 10.1385/ENDO:27:1:037 16077169

[B157] Rawls J. F. (2007). Enteric Infection and Inflammation Alter Gut Microbial Ecology. Cell Host Microbe 2, 73–74. 10.1016/j.chom.2007.07.006 18005720 PMC4843771

[B158] Rawls J. F. Mahowald M. A. Ley R. E. Gordon J. I. (2006). Reciprocal Gut Microbiota Transplants from Zebrafish and Mice to Germ-free Recipients Reveal Host Habitat Selection. Cell 127, 423–433. 10.1016/j.cell.2006.08.043 17055441 PMC4839475

[B159] Ray A. k. Ghosh K. Ringø E. (2012). Enzyme-producing Bacteria Isolated from Fish Gut: a Review. Aquac. Nutr. 18, 465–492. 10.1111/j.1365-2095.2012.00943.x

[B160] Ridlon J. M. Ikegawa S. Alves J. M. P. Zhou B. Kobayashi A. Iida T. (2013). Clostridium Scindens: a Human Gut Microbe with a High Potential to Convert Glucocorticoids into Androgens. J. Lipid Res. 54, 2437–2449. 10.1194/jlr.M038869 23772041 PMC3735941

[B161] Roeselers G. Mittge E. K. Stephens W. Z. Parichy D. M. Cavanaugh C. M. Guillemin K. (2011). Evidence for a Core Gut Microbiota in the Zebrafish. ISME J. 5, 1595–1608. 10.1038/ismej.2011.38 21472014 PMC3176511

[B162] Romero J. Navarrete P. (2006). 16S rDNA-Based Analysis of Dominant Bacterial Populations Associated with Early Life Stages of Coho Salmon (*Oncorhynchus kisutch*). Microb. Ecol. 51, 422–430. 10.1007/s00248-006-9037-9 16598631

[B163] Roosterman D. Goerge T. Schneider S. W. Bunnett N. W. Steinhoff M. (2006). Neuronal Control of Skin Function: The Skin as a Neuroimmunoendocrine Organ. Physiol. Rev. 86, 1309–1379. 10.1152/physrev.00026.2005 17015491

[B164] Roshchina V. V. (2010). “Evolutionary Considerations of Neurotransmitters in Microbial, Plant, and Animal Cells,” in Microbial Endocrinology: Interkingdom Signaling in Infectious Disease and Health. Editors Lyte M. Freestone P. P. E. (New York, NY: Springer), 17–52. 10.1007/978-1-4419-5576-0_2

[B165] Roughgarden J. Gilbert S. F. Rosenberg E. Zilber-Rosenberg I. Lloyd E. A. (2018). Holobionts as Units of Selection and a Model of Their Population Dynamics and Evolution. Biol. Theory 13, 44–65. 10.1007/s13752-017-0287-1

[B166] Round J. L. Mazmanian S. K. (2009). The Gut Microbiome Shapes Intestinal Immune Responses during Health and Disease. Nat. Rev. Immunol. 9, 313–323. 10.1038/nri2515 19343057 PMC4095778

[B167] Sánchez M. I. Pons I. Martínez-Haro M. Taggart M. A. Lenormand T. Green A. J. (2016). When Parasites Are Good for Health: Cestode Parasitism Increases Resistance to Arsenic in Brine Shrimps. PLOS Pathog. 12, e1005459. 10.1371/journal.ppat.1005459 26938743 PMC4777290

[B168] Sandhu K. V. Sherwin E. Schellekens H. Stanton C. Dinan T. G. Cryan J. F. (2017). Feeding the Microbiota-Gut-Brain axis: Diet, Microbiome, and Neuropsychiatry. Transl. Res. 179, 223–244. 10.1016/j.trsl.2016.10.002 27832936

[B169] Sarkodie E. K. Zhou S. Baidoo S. A. Chu W. (2019). Influences of Stress Hormones on Microbial Infections. Microb. Pathog. 131, 270–276. 10.1016/j.micpath.2019.04.013 30981718

[B170] Semova I. Carten J. D. Stombaugh J. Mackey L. C. Knight R. Farber S. A. (2012). Microbiota Regulate Intestinal Absorption and Metabolism of Fatty Acids in the Zebrafish. Cell Host Microbe 12, 277–288. 10.1016/j.chom.2012.08.003 22980325 PMC3517662

[B171] Senthilkumaran B. Okuzawa K. Gen K. Kagawa H. (2001). Effects of Serotonin, GABA and Neuropeptide Y on Seabream Gonadotropin Releasing Hormone Release *In Vitro* from Preoptic-Anterior Hypothalamus and Pituitary of Red Seabream, *Pagrus major* . J. Neuroendocrinol. 13, 395–400. 10.1046/j.1365-2826.2001.00645.x 11328447

[B172] Sheng Y. Ren H. Limbu S. M. Sun Y. Qiao F. Zhai W. (2018). The Presence or Absence of Intestinal Microbiota Affects Lipid Deposition and Related Genes Expression in Zebrafish (*Danio rerio*). Front. Microbiol. 9, 1124. 10.3389/fmicb.2018.01124 29896183 PMC5987169

[B173] Siders A. r. (2019). Adaptive Capacity to Climate Change: A Synthesis of Concepts, Methods, and Findings in a Fragmented Field. WIREs Clim. Change 10, e573. 10.1002/wcc.573

[B174] Silva F. C. de P. Nicoli J. R. Zambonino-Infante J. L. Kaushik S. Gatesoupe F.-J. (2011). Influence of the Diet on the Microbial Diversity of Faecal and Gastrointestinal Contents in Gilthead Sea Bream (*Sparus aurata*) and Intestinal Contents in Goldfish (*Carassius auratus*). FEMS Microbiol. Ecol. 78, 285–296. 10.1111/j.1574-6941.2011.01155.x 21692817

[B214] Skrodenyte V. A. Sruoga A. Butkauskas D. Skrupskelis K. (2008). Phylogenetic Analysis of Intestinal Bacteria of Freshwater Salmon *Salmo salar* and Sea Trout *Salmo trutta trutta* and Diet. Fish. Sci. 74, 1307–1314. 10.1111/j.1444-2906.2008.01656.x

[B175] Slominski A. T. Zmijewski M. A. Skobowiat C. Zbytek B. Slominski R. M. Steketee J. D. (2012). Sensing the Environment: Regulation of Local and Global Homeostasis by the Skin Neuroendocrine System. Adv. Anat. Embryol. Cell Biol. 212, 1–115. 10.1007/978-3-642-19683-6_1 PMC3422784 22894052

[B176] Slominski A. Wortsman J. Luger T. Paus R. Solomon S. (2000). Corticotropin Releasing Hormone and Proopiomelanocortin Involvement in the Cutaneous Response to Stress. Physiol. Rev. 80, 979–1020. 10.1152/physrev.2000.80.3.979 10893429

[B177] Smriga S. Sandin S. A. Azam F. (2010). Abundance, Diversity, and Activity of Microbial Assemblages Associated with Coral Reef Fish Guts and Feces. FEMS Microbiol. Ecol. 73, 31–42. 10.1111/j.1574-6941.2010.00879.x 20455942

[B178] Somoza G. M. Peter R. E. (1991). Effects of Serotonin on Gonadotropin and Growth Hormone Release from *In Vitro* Perifused Goldfish Pituitary Fragments. General Comp. Endocrinol. 82, 103–110. 10.1016/0016-6480(91)90301-L 1874380

[B179] Soriano E. L. Ramírez D. T. Araujo D. R. Gómez-Gil B. Castro L. I. Sánchez C. G. (2018). Effect of Temperature and Dietary Lipid Proportion on Gut Microbiota in Yellowtail Kingfish *Seriola lalandi* Juveniles. Aquaculture 497, 269–277. 10.1016/j.aquaculture.2018.07.065

[B180] Spor A. Koenig J. E. Scalfone N. Fricker A. D. Stombaugh J. Knight R. (2011). Succession of Microbial Consortia in the Developing Infant Gut Microbiome. PNAS 108, 4578–4585. 10.1073/pnas.1000081107 20668239 PMC3063592

[B181] Steele J. A. Countway P. D. Xia L. Vigil P. D. Beman J. M. Kim D. Y. (2011). Marine Bacterial, Archaeal and Protistan Association Networks Reveal Ecological Linkages. ISME J. 5, 1414–1425. 10.1038/ismej.2011.24 21430787 PMC3160682

[B182] Sternberg E. M. (2006). Neural Regulation of Innate Immunity: a Coordinated Nonspecific Host Response to Pathogens. Nat. Rev. Immunol. 6, 318–328. 10.1038/nri1810 16557263 PMC1783839

[B183] Sylvain F.-É. Holland A. Audet-Gilbert É. Luis Val A. Derome N. (2019). Amazon Fish Bacterial Communities Show Structural Convergence along Widespread Hydrochemical Gradients. Mol. Ecol. 28, 3612–3626. 10.1111/mec.15184 31325401

[B184] Talwar C. Nagar S. Lal R. Negi R. K. (2018). Fish Gut Microbiome: Current Approaches and Future Perspectives. Indian J. Microbiol. 58, 397–414. 10.1007/s12088-018-0760-y 30262950 PMC6141390

[B185] Tan J. Wang C. Wu Y. Wei H. Sun H. Peng J. (2019). Logistic Regression Analysis Factors Affecting Sperm Motility and Abnormal Sperm Morphology in Boars. Animals 9, 1004. 10.3390/ani9121004 PMC6941152 31756982

[B186] Tecott L. H. Abdallah L. (2003). Mouse Genetic Approaches to Feeding Regulation: Serotonin 5-HT2C Receptor Mutant Mice. CNS Spectrums 8, 578–588. 10.1017/S109285290001885X 12907921

[B187] Thompson F. L. Iida T. Swings J. (2004). Biodiversity of Vibrios. Microbiol. Mol. Biol. Rev. 68 (3), 403–431. 10.1128/MMBR.68.3.403-431.2004 15353563 PMC515257

[B188] Tollefson J. (2018). IPCC Says Limiting Global Warming to 1.5 °C Will Require Drastic Action. Nature 562, 172–173. 10.1038/d41586-018-06876-2 30301994

[B189] Trudeau V. L. Kah O. Chang J. P. Sloley B. D. Dubourg P. Fraser E. J. (2000). The Inhibitory Effects of (Gamma)-aminobutyric Acid (GABA) on Growth Hormone Secretion in the Goldfish Are Modulated by Sex Steroids. J. Exp. Biol. 203, 1477–1485. 10.1242/jeb.203.9.1477 10751163

[B190] Trudeau V. L. Sloley B. D. Wong A. O. L. Peter R. E. (1993). Interactions of Gonadal Steroids with Brain Dopamine and Gonadotropin-Releasing Hormone in the Control of Gonadotropin-II Secretion in the Goldfish. General Comp. Endocrinol. 89, 39–50. 10.1006/gcen.1993.1007 8094059

[B191] Trudeau V. (1997). Neuroendocrine Regulation of Gonadotrophin II Release and Gonadal Growth in the Goldfish, *Carassius auratus* . Rev. reproduction 2, 55–68. 10.1530/revreprod/2.1.55 9414466

[B192] Tsavkelova E. A. Klimova S. Yu. Cherdyntseva T. A. Netrusov A. I. (2006). Hormones and Hormone-like Substances of Microorganisms: A Review. Appl. Biochem. Microbiol. 42, 229–235. 10.1134/S000368380603001X 16878539

[B193] Tsuchiya C. Sakata T. Sugita H. (2008). Novel Ecological Niche of Cetobacterium Somerae, an Anaerobic Bacterium in the Intestinal Tracts of Freshwater Fish. Lett. Appl. Microbiol. 46, 43–48. 10.1111/j.1472-765X.2007.02258.x 17944860

[B194] Urakawa H. Kita-Tsukamoto K. Ohwada K. Y. (1999). Microbial Diversity in Marine Sediments from Sagami Bay and Tokyo Bay, Japan, as Determined by 16S rRNA Gene analysisThe DDBJ Accession Numbers for the Sequences Reported in This Paper Are AB022607–Ab022642. Microbiology 145, 3305–3315. 10.1099/00221287-145-11-3305 10589740

[B195] Valcarce D. G. Pardo M. Á. Riesco M. F. Cruz Z. Robles V. (2015). Effect of Diet Supplementation with a Commercial Probiotic Containing Pediococcus Acidilactici (Lindner, 1887) on the Expression of Five Quality Markers in Zebrafish (*Danio rerio* (Hamilton, 1822)) Testis. J. Appl. Ichthyology 31, 18–21. 10.1111/jai.12731

[B196] van de Wetering M. Sancho E. Verweij C. de Lau W. Oving I. Hurlstone A. (2002). The β-Catenin/TCF-4 Complex Imposes a Crypt Progenitor Phenotype on Colorectal Cancer Cells. Cell 111, 241–250. 10.1016/S0092-8674(02)01014-0 12408868

[B197] Vandenberghe J. Thompson F. L. Gomez-Gil B. Swings J. (2003). Phenotypic Diversity Amongst Vibrio Isolates from Marine Aquaculture Systems. Aquaculture 219, 9–20. 10.1016/S0044-8486(02)00312-5

[B198] Wammes L. J. Mpairwe H. Elliott A. M. Yazdanbakhsh M. (2014). Helminth Therapy or Elimination: Epidemiological, Immunological, and Clinical Considerations. Lancet Infect. Dis. 14, 1150–1162. 10.1016/S1473-3099(14)70771-6 24981042

[B199] Wang N. Milla S. Mandiki S. N. M. Kestemont P. (2009). Corticosteroids: Friends or Foes of Teleost Fish Reproduction? Comp. Biochem. Physiology Part A Mol. Integr. Physiology 153, 242–251. 10.1016/j.cbpa.2009.02.027 19254778

[B200] Wang X. Sun G. Feng T. Zhang J. Huang X. Wang T. (2019). Sodium Oligomannate Therapeutically Remodels Gut Microbiota and Suppresses Gut Bacterial Amino Acids-Shaped Neuroinflammation to Inhibit Alzheimer’s Disease Progression. Cell Res. 29, 787–803. 10.1038/s41422-019-0216-x 31488882 PMC6796854

[B201] Wang Y. Ge W. (2003a). Gonadotropin Regulation of Follistatin Expression in the Cultured Ovarian Follicle Cells of Zebrafish, *Danio rerio* . General Comp. Endocrinol. 134, 308–315. 10.1016/S0016-6480(03)00275-2 14636638

[B202] Wang Y. Ge W. (2003b). Involvement of Cyclic Adenosine 3′,5′-Monophosphate in the Differential Regulation of Activin βA and βB Expression by Gonadotropin in the Zebrafish Ovarian Follicle Cells. Endocrinology 144, 491–499. 10.1210/en.2002-220734 12538609

[B203] Wang Y. Ge W. (2003c). Spatial Expression Patterns of Activin and its Signaling System in the Zebrafish Ovarian Follicle: Evidence for Paracrine Action of Activin on the Oocytes1. Biol. Reproduction 69, 1998–2006. 10.1095/biolreprod.103.020826 12930712

[B204] Watts J. E. M. McDonald R. Daniel R. Schreier H. J. (2013). Examination of a Culturable Microbial Population from the Gastrointestinal Tract of the Wood-Eating Loricariid Catfish Panaque Nigrolineatus. Diversity 5, 641–656. 10.3390/d5030641

[B205] Won E. Borski R. (2013). Endocrine Regulation of Compensatory Growth in Fish. Front. Endocrinol. 4, 74. 10.3389/fendo.2013.00074 PMC3696842 23847591

[B206] Wu J.-M. Liu R.-H. (2012). Thin Stillage Supplementation Greatly Enhances Bacterial Cellulose Production by Gluconacetobacter Xylinus. Carbohydr. Polym. 90, 116–121. 10.1016/j.carbpol.2012.05.003 24751018

[B207] Yan N. Chen X. (2015). Sustainability: Don’t Waste Seafood Waste. Nature 524, 155–157. 10.1038/524155a 26268177

[B208] Yukgehnaish K. Kumar P. Sivachandran P. Marimuthu K. Arshad A. Paray B. A. (2020). Gut Microbiota Metagenomics in Aquaculture: Factors Influencing Gut Microbiome and its Physiological Role in Fish. Rev. Aquac. 12, 1903–1927. 10.1111/raq.12416

[B209] Zhou Z. Liu Y. Shi P. He S. Yao B. Ringø E. (2009). Molecular Characterization of the Autochthonous Microbiota in the Gastrointestinal Tract of Adult Yellow Grouper (Epinephelus Awoara) Cultured in Cages. Aquaculture 286, 184–189. 10.1016/j.aquaculture.2008.10.002

[B210] Zieba D. A. Amstalden M. Williams G. L. (2005). Regulatory Roles of Leptin in Reproduction and Metabolism: A Comparative Review. Domest. Anim. Endocrinol. 29, 166–185. 10.1016/j.domaniend.2005.02.019 15927772

