# Supplementary material for: Tenets in Microbial Endocrinology: A New Vista in Teleost Reproduction
Source: Front Physiol. 2022 Aug 12;13:871045. doi: 10.3389/fphys.2022.871045 (PMC9411670; doi:10.3389/fphys.2022.871045)
Supplement: Supplementary file 1 [file Table1.DOCX]

**Table 1. Microbial composition of gut microbiota in herbivore, carnivore and omnivore fish**

| **Bacterial species** | **Fish species** | **References** |
| --- | --- | --- |
| **Herbivores** | | |
| *Clostridium sp., Eubacterium desmolans, Papillibacter* | Butterfish (*Odax pullus),*  Marblefish (*Aplodactylus arctidens*) | Clements et al. (2007) |
| *Vibrio sp., Photobacterium, Bacteroidetes,*  **non-vibrio** *Proteobacteria, Firmicutes* | Parrotfish (*Chlorurus sordidus*),  Surgeonfish (*Acanthurus nigricans*) | Smriga et al. (2010) |
| *Clostridium sp.* | Silver drummer (*Kyphosus sydneyanus)* | Moran et al. (2005) |
| *Epulopiscium* | Surgeonfish (*Acanthurus sp*.) | Miyake et al. (2015) |
| **Zooplanktivores** | | |
| *Vibrionaceae, Pasteurellaceae, Vibrio*  *harveyi, Shewanella sp., Endozoicomonas sp.* | Cardinalfish, Damselfish | Parris et al. (2016) |
| *Pseudomonas, Alteromonas, Psychrobacter* | Herring (*Clupea harengis*) | Curson et al. (2010 |
| *Psychrobacter, Vibrio sp., Shewanella* | Atlantic mackerel (*Scomber scombrus)* | Svanevik & Lunestad (2011) |
| **Carnivores** | | |
| *Clostridium perfringens, Vibrio sp.* | Atlantic cod (*Gadus morhua*) | Star et al. (2013) |
| *Pseudomonas sp.* | Gilthead seabream (*Sparus aurata)* | Floris et al. (2013) |
| *Bacillus, Vibrio, Delftia, Psychroacter,*  *Acinetobacter, Pseudomona* | Grouper (*Epinephelus coioides*) | Sun et al. (2009) |
| *Aeromonas sobria, Pseudomonas* | Sea trout (*Salmo trutta trutta*) | Skrodenyt et al. (2008) |
| *Escherichia coli* | Speckled trout (*Cynoscion nebulosus*) | Ransom (2008) |
| **Omnivores** | | |
| *Clostridium, Mycoplasma, Photobacterium,*  *Propionibacterium, Staphylococcus,*  *Pseudomonas, Corynebacterium* | Pinfish (*Lagodon rhomboids)* | Givens et al. (2015) |
| *Mycoplasma* | Long-jawed mudsucker (*Gillichthys mirabilis*) | Bano et al. (2007) |

**Table 2. Probiotics used in aquaculture practices and their effect on their host**

| **Probiotic strain** | **Host species** | **Effect** | **Reference** |
| --- | --- | --- | --- |
| *Vibrio fluviales* A3-47S,  *Aeromonas hydrophila* A3-51,  *Carnobacterium sp*. BA211,  *Micrococcus luteus* A1-6 | *Oncorhynchus*  *mykiss* | Immune stimulation and improved survival after challenge with *Aeromonas salmonicida* | Irianto & Austin  (2002) |
| *Lactobacillus rhamnosus* ATCC 53103 | *Oncorhynchus*  *mykiss* | Immune stimulation and improved survival after  challenge with *Aeromonas salmonicida* | Nikoskelainen et al.  (2001, 2003) |
| *Lactococcus lactis CECT 539* | *Scophthalmus*  *maximus* | Immune stimulation | Villamil et al. (2002 |
| *Lactobacillus rhamnosus* JCM 1136 | *Oncorhynchus*  *mykiss* | Immune stimulation | Panigrahi et al. (2004) |
| *Lactobacillus delbriieckii* CECT 287,  *Bacillus subtilis* CECT 35 | *Sparus aurata* | Immune stimulation | Salinas et al. (2005) |
| *Aeromonas sobria* GC2 | *Oncorhynchus*  *mykiss* | Immune stimulation and improved survival after  challenge with *Lactococcus garvieae* and  *Streptococcus iniae* | Brunt & Austin (2005) |
| *Bacillus subtilis*, *Lactobacillus acidophilus, Clostridium butyricum, Saccharomyces*  *cerevisiae* | *Paralichthys*  *olivaceus* | Immune stimulation and improved survival after  challenge with *Vibrio anguillarum* | Taoka et al. (2006) |
| *Carnobacterium maltaromaticum* B26  *Carnobacterium divergens* B33 | *Oncorhynchus*  *mykiss* | Immune stimulation and improved survival after  challenge with *Aeromonas salmonicida* and *Yersinia*  *ruckeri*. Expression of cytokine genes | Kim & Austin  (2006) |
| *Lactobacillus rhamnosus* ATCC 53103 | *Oreochromis*  *niloticus* | Immune stimulation and improved survival after  challenge with *Edwardsiella tarda* | Pirarat et al. (2006) |
| *Lactobacillus rhamnosus* ATCC 53103  *Bacillus subtilis* | *Oncorhynchus*  *mykiss* | Immune stimulation and expression of cytokine  genes | Panigrahi et al. (2007) |
| *Lactobacillus sakei* CLFP 202,  *Lactococcus lactis* CLFP 100  *Leuconostoc mesenteroides* CLFP 196 | *Oncorhynchus*  *mykiss,*  *Salmo trutta* | Immune stimulation and improved survival  after challenge with *Aeromonas salmonicida* | Balca´ zar et al.  (2006) |
| *Lactobacillus plantarum* CLFP 238  *Leuconostoc mesenteroides* CLFP 196 | *Oncorhynchus*  *mykiss* | Competitive exclusion and improved survival  after challenge with *Lactococcus garvieae* | Vendrell et al. (2007) |
